# Tissue MicroRNAs as Predictors of Outcome in Patients with Metastatic Colorectal Cancer Treated with First Line Capecitabine and Oxaliplatin with or without Bevacizumab

**DOI:** 10.1371/journal.pone.0109430

**Published:** 2014-10-15

**Authors:** Mogens K. Boisen, Christian Dehlendorff, Dorte Linnemann, Boye S. Nielsen, Jim S. Larsen, Kell Østerlind, Svend E. Nielsen, Line S. Tarpgaard, Camilla Qvortrup, Per Pfeiffer, Niels H. Holländer, Nina Keldsen, Torben F. Hansen, Brita B. Jensen, Estrid V. S. Høgdall, Benny V. Jensen, Julia S. Johansen

**Affiliations:** 1 Department of Oncology, Herlev University Hospital, Herlev, Denmark; 2 Statistics, Bioinformatics and Registry, Danish Cancer Society Research Center, Copenhagen, Denmark; 3 Department of Pathology, Herlev University Hospital, Herlev, Denmark; 4 Bioneer, Hørsholm, Denmark; 5 Department of Oncology, Roskilde Hospital, Roskilde, Denmark; 6 Department of Oncology, Rigshospitalet, Copenhagen, Denmark; 7 Department of Oncology and Palliation, Hillerød Hospital, Hillerød, Denmark; 8 Department of Oncology, Odense University Hospital, Odense, Denmark; 9 Department of Oncology, Næstved Hospital, Næstved, Denmark; 10 Department of Oncology, Herning Hospital, Herning, Denmark; 11 Department of Oncology, Vejle Hospital, Vejle, Denmark; 12 Department of Medicine, Queen Ingrid Hospital, Nuuk, Greenland; 13 Department of Oncology, Sydvestjysk Hospital, Esbjerg, Denmark; 14 Department of Medicine, Herlev University Hospital, Herlev, Denmark; Saint Louis University, United States of America

## Abstract

**Purpose:**

We tested the hypothesis that expression of microRNAs (miRNAs) in cancer tissue can predict effectiveness of bevacizumab added to capecitabine and oxaliplatin (CAPEOX) in patients with metastatic colorectal cancer (mCRC).

**Experimental Design:**

Patients with mCRC treated with first line CAPEOX and bevacizumab (CAPEOXBEV): screening (n = 212) and validation (n = 121) cohorts, or CAPEOX alone: control cohort (n = 127), were identified retrospectively and archival primary tumor samples were collected. Expression of 754 miRNAs was analyzed in the screening cohort using polymerase chain reaction (PCR) arrays and expression levels were related to time to disease progression (TTP) and overall survival (OS). Significant miRNAs from the screening study were analyzed in all three cohorts using custom PCR arrays. *In situ* hybridization (ISH) was done for selected miRNAs.

**Results:**

In the screening study, 26 miRNAs were significantly correlated with outcome in multivariate analyses. Twenty-two miRNAs were selected for further study. Higher miR-664-3p expression and lower miR-455-5p expression were predictive of improved outcome in the CAPEOXBEV cohorts and showed a significant interaction with bevacizumab effectiveness. The effects were strongest for OS. Both miRNAs showed high expression in stromal cells. Higher expression of miR-196b-5p and miR-592 predicted improved outcome regardless of bevacizumab treatment, with similar effect estimates in all three cohorts.

**Conclusions:**

We have identified potentially predictive miRNAs for bevacizumab effectiveness and additional miRNAs that could be related to chemotherapy effectiveness or prognosis in patients with mCRC. Our findings need further validation in large cohorts, preferably from completed randomized trials.

## Introduction

Colorectal cancer (CRC) is a leading cause of cancer-related mortality worldwide [1]. Most deaths occur as a result of the development of metastatic CRC (mCRC). Standard of care for patients with mCRC who cannot undergo radical resection of metastases is system chemotherapy with or without a targeted agent [2]. Bevacizumab is a monoclonal antibody that binds the ligand ‘vascular endothelial growth factor’ (VEGF-A) and thereby inhibits the ability of cancers to produce new blood vessels from existing vessels, a process called angiogenesis. Bevacizumab has demonstrated efficacy in patients with mCRC when used in combination with standard chemotherapy but the benefit is modest when used unselectively and bevacizumab adds significant toxicity and cost to the treatment [3]–[7]. Therefore, the identification of predictive biomarkers for bevacizumab has become a major goal of biomarker research in patients with mCRC. Due to its widespread adoption as a standard first- or second line treatment [8], the ability to individualize bevacizumab treatment would have a great impact on clinical practice. Numerous studies have investigated potential biomarkers in the form of RNA, DNA, or protein [9], [10]. None has made it into the clinic. Currently, no commercially available test can identify patients who will benefit from bevacizumab.

MicroRNAs (miRNAs) are small, ∼22 nucleotides long, non-coding RNAs involved in post-transcriptional regulation of gene expression. They have been intensely investigated as biomarkers in patients with cancer because their expression levels are dysregulated in cancer cells, they can influence cancer behavior, and they are relatively resistant to degradation in commonly used sampling media [11]–[16]. Several studies have identified dysregulation of miRNAs in CRC tumor tissue and in blood samples from patients with CRC; and some of the identified miRNAs were also associated with prognostic factors like depth of invasion, stage, and lymph node metastases [17]. Furthermore, important molecular features in CRC such as micro-satellite instability (MSI) and *BRAF* mutational status have been shown to be associated with distinct miRNA expression patterns [18]. Hence, there is a strong rationale for investigating the potential utility of miRNA expression as a predictive or prognostic biomarker in patients with CRC. To date, no published study has explored the predictive value of miRNA expression for bevacizumab effectiveness in a comprehensive manner.

We aimed to identify miRNAs that were predictive of outcome in patients with mCRC treated with first line capecitabine and oxaliplatin with and without bevacizumab (CAPEOXBEV/CAPEOX) and to identify which of these miRNAs could be predictive for the effect of bevacizumab-addition to chemotherapy.

## Methods

### Study design

A screening study using an array approach was performed on primary CRC tissue samples from patients treated with CAPEOXBEV (screening cohort) to identify candidate miRNAs with expression levels related to outcome. Thereafter, the expression levels of the identified candidate miRNAs were measured using a more precise method with duplicate determinations in three cohorts: a subgroup of the screening cohort; a validation cohort, which was an independent group of patients treated with CAPEOXBEV; and a control cohort, consisting of patients treated with CAPEOX alone.

### Patients, data extraction, and end points

The BETmiRC (Bevacizumab Tissue microRNAs in Colorectal cancer) study retrospectively included patients with mCRC treated with first line CAPEOXBEV in 10 Danish hospitals from 2006 to 2011,and patients treated with first line CAPEOX at Herlev Hospital or in a randomized study from 2003 to 2006 [19], before bevacizumab was approved, as previously described [20].The end points time to disease progression (TTP) and overall survival (OS) were measured from initiation of treatment to disease progression or death from any cause, respectively (detailed definition in File S1). Vital status was updated July 5, 2013.

### Tissue samples

Formalin-fixed paraffin-embedded (FFPE) tissue blocks containing samples from primary tumors were retrieved using the National Pathology Registry. Control samples from patients resected for inflammatory bowel disease were also included. An experienced gastro-intestinal pathologist (DL) selected which tissue blocks to retrieve and scored the blocks for tumor cell percentage. Three 10-µm sections were cut from each block without micro- or macro-dissection and the sections were placed in sterile Eppendorf tubes. All tissue samples were collected prior to any systemic treatment or radiotherapy.

### MiRNA expression analysis

RNA was purified using the miRNeasy FFPE Kit (Qiagen, Hilden, Germany) using the manufacturer's instructions. The purification order was randomized for the validation and control cohort samples. The non-human miRNA ath-miR-159a was added to each sample before cDNA synthesis as a spike-in control.

The TaqMan Human MicroRNA array A and B Cards Set v3.0 (Applied Biosystems) was used to quantify expression of 754 human miRNAs with single determinations in the screening study. In the subsequent study of the reduced screening-, validation-, and control cohorts, miRNA expression was measured using TaqMan Custom LDA cards (Applied Biosystems) profiling 22 selected miRNAs in duplicate with 8 samples on each card. The 22 miRNAs were selected from the screening study and the micro-fluidic cards were pre-configured from the manufacturer according to our specifications. Samples were analyzed in a randomized order on the Custom LDA cards.

The instructions and reagents from the manufacturer were used in all steps (https://www.products.appliedbiosystems.com). All RNA purification- and miRNA expression studies were performed by AROS Applied Biotechnology (Aarhus, Denmark). The company was blinded to all clinical information.

### MiRNA *in situ* hybridization

*In situ* hybridization (ISH) was performed using double-FAM (carboxyfluorescein) labeled locked nucleic acid (LNA [21]) probes (Exiqon, Vedbæk, Denmark) for miR-185-5p, miR-455-5p, miR-592, miR-664-3p, miR-21-5p, and miR-126-3p, as previously described [22]. All ISH studies were performed by Bioneer (Hørsholm, Denmark).

### Statistical analysis

No sample size calculation was done prior to study initiation. We aimed for the greatest sample size possible and equal sizes of the three cohorts.

#### MiRNA expression analyses – screening study

Raw cycle threshold (C_t_) for each miRNA was checked for outliers and data were corrected using spike-in values. In a univariate selection method, the expression of each miRNA was related to TTP and OS using a Cox proportional hazards (CPH) model [23], [24]. Candidate miRNAs were included in a multivariate CPH model adjusted for age, sex, histology, number of metastatic sites, primary tumor location, and prior adjuvant treatment, which was simplified using a backwards elimination procedure based upon Akaike's Information Criterion [25]. The analysis was then repeated for data sets normalized using quantile- and mean normalization. Finally, 22 miRNAs were selected for further study based primarily on their performance in the multivariate analyses. The number of miRNAs to include in the second study was chosen pragmatically as it allowed for duplicate measurements on the custom platform.

#### MiRNA expression analyses – screening-, validation-, and control cohorts

Mean C_t_ of the duplicate measurements was calculated and transformed to 40-C_t_. If one of the two measurements was undetermined, the C_t_ of the other measurement was used. In each of the three cohorts, expression of the 22 miRNAs was related to TTP and OS using CPH models with adjustment for age, sex, primary tumor location, prior adjuvant treatment, and number of metastatic sites. Results were reported as hazard ratios (HR) per inter-quartile range increase in expression level with 95% confidence intervals (CI).The possible interaction between miRNA expression level and bevacizumab treatment effect was tested in the three cohorts combined using a likelihood ratio test.

Since outcome for the patients treated with bevacizumab differed greatly depending on primary tumor location [20], we also performed the interaction analyses for proximal and distal primary cancers separately.

*P*<0.05 was considered statistically significant and no formal corrections for multiple comparisons were made. The statistical software packages R [26] (www.r-project.org) and GraphPad Prism 5 (GraphPad Software, Inc) were used for all analyses.

### Target prediction

*In silico* predicted gene targets for selected miRNAs were identified using the open-access DIANA-microT-CDS tool (http://www.microrna.gr/microT-CDS) [27].

### Ethics

The study was approved by the Regional Scientific Ethics Committee of the Capital Region of Denmark (http://www.regionh.dk/vek, approval number: H-1-2010-081). Since this retrospective study would not have any influence on treatment and since most of the participants were deceased, written consent was not obtained, and this was approved by the ethics committee.

Reporting of the results was prepared according to the REMARK guidelines [28], [29].

Further details are described in File S1.

## Results

MiRNA expression was measured in 460 FFPE samples. The number of samples in each cohort was: screening cohort = 212, reduced screening cohort = 155, validation cohort = 121, and control cohort = 127 (Figure S1 in File S1).

The patients included in the reduced screening- and the validation miRNA cohorts differed from the patients not included. They were more likely to have: resected primary tumor, a single metastatic site, performance status 0, and prior adjuvant therapy. They also experienced a longer TTP and OS (Table 1). The patients in the control cohort were similar to the patients not included except for them being more likely to have had the primary tumor resected.

**Table 1:** Patient and sample characteristics.

	Clinical CAPEOXBEV	Clinical CAPEOX	Screening cohort	Screening cohort	Validation cohort	CAPEOXBEV	CAPEOX cohort	CAPEOX
	cohort	cohort	(LDA A+B card)	(cLDA)	(cLDA)	included vs. not-included	(cLDA)	included vs. not-included
						*P*		*P*
Treatment start years, range	2006–2011	2003–2006	2006–2010	2006–2010	2007–2011		2003–2006	
Number of patients	623	211	203	155	121		127	
Age, median (range)	65 (22–84)	63 (36–85)	65 (37–84)	65 (40–84)	67 (22–82)	0.27	62 (36–82)	0.24
Sex, female (%)	281 (45%)	77 (36%)	111 (55%)	83 (54%)	40 (33%)	0.75	45 (35%)	0.66
Primary tumor location						0.15		0.45
Cecum, ascending colon	133 (21%)	37 (18%)	52 (26%)	38 (25%)	23 (19%)		26 (20%)	
Right flexure, transverse colon	60 (10%)	16 (8%)	15 (7%)	11 (7%)	7 (6%)		8 (6%)	
Left flexure, descending colon	33 (5%)	12 (6%)	9 (4%)	6 (4%)	7 (6%)		9 (7%)	
Sigmoid colon	192 (31%)	62 (29%)	70 (34%)	56 (36%)	38 (31%)		37 (29%)	
Rectum	205 (33%)	84 (40%)	57 (28%)	44 (28%)	46 (38%)		47 (37%)	
Primary tumor resected	319 (51%)	157 (74%)	186 (92%)	148 (95%)	84 (69%)	**<0.0001**	116 (91%)	**<0.0001**
Number of metastatic sites						**0.004**		1.00
1	198 (32%)	71 (34%)	74 (36%)	63 (41%)	42 (35%)		42 (33%)	
>1	425 (68%)	140 (66%)	129 (64%)	92 (59%)	79 (65%)		85 (67%)	
No. of first line cycles, median (range)	7 (1–46)	6 (1–16)*	7 (1–46)	7 (1–46)	7 (1–23)	0.25	6 (1–15)*	0.92
Treated with irinotecan	357 (58%)*	105 (64%)*	117 (58%)	95 (61%)	76 (63%)	0.05	64 (72%)	0.29
Performance status						**0.04**		0.32
0	260 (61%)	81 (50%)	93 (61%)	75 (65%)	60 (71%)		48 (48%)	
1	138 (32%)	66 (41%)	52 (34%)	37 (32%)	19 (22%)		39 (39%)	
≥2	27 (6%)	15 (9%)	8 (5%)	3 (3%)	6 (7%)		12 (12%)	
Missing	198	49	50	40	36		28	
Prior adjuvant treatment, no. (%)	57 (9%)	37 (18%)	28 (14%)	22 (14%)	13 (11%)	**0.008**	26 (20%)	0.20
TTP events				130 (84%)	98 (81%)		115 (91%)	
Median time to disease progression	8.6 mo	7.6 mo	9.2 mo	9.5 mo	8.3 mo	**0.04**	8.4 mo	0.27
OS events				130 (84%)	93 (77%)		125 (98%)	
Median overall survival	16.1 mo	15.2 mo	19.0 mo	25.0 mo	21.9 mo	**<0.0001**	16.4 mo	0.12
Sample type								
Resection	–	–	–	146 (94%)	83 (69%)		115 (91%)	
Biopsy	–	–	–	9 (6%)	38 (31%)		12 (9%)	
Tumor cell percentage, median (range)	–	–	–	30 (2–70)	30 (5–70)		30 (5–70)	
cLDA mean C_t_, median (1./3. quartile)	–	–	–	19.9 (19.5/20.9)	20.8 (20.0/22.0)		20.7 (20.3/21.7)	

* Data were missing for some patients; ranges and percentages are based on the patients with available data.

### Screening study

Nine samples were identified as outliers based on a low number of miRNAs detected and these were excluded, leaving 203 samples for outcome calculations. Twenty-six miRNAs were associated with TTP or OS in multivariate analysis using raw-, quantile-normalized, or mean-normalized expression data (Table S1 in File S1). Twenty-two of these were selected for further study: miR-1, miR-15a-5p, miR-17-3p, miR-22-3p, miR-29b-3p, miR-145-3p, miR-155-5p, miR-185-5p, miR-193b-5p, miR-196b-5p, miR-204-5p, miR-214-5p, miR-338-3p, miR-382-5p, miR-449a, miR-455-5p, miR-497-5p, miR-501-5p, miR-545-3p, miR-552-3p, miR-592 and miR-664-3p.

### Focused miRNA panel – miRNAs associated with TTP

Eleven miRNAs were significantly associated with TTP in either the screening- or the validation cohort, but not in both cohorts and no significant interactions were found between miRNA expression and bevacizumab effect (Table 2).

**Table 2:** Focused miRNA panel – miRNAs significantly associated with time to disease progression.

	Screening CAPEOXBEV cohort			Validation CAPEOXBEV cohort			Combined CAPEOXBEV cohort			Control CAPEOX cohort			Interaction^a^		
	n = 155			n = 121			n = 276			n = 127			all	distal	proximal
miRNA	HR^b^	95% CI	*P*	HR	95% CI	*P*	HR	95% CI	*P*	HR	95% CI	*P*	*P*	*P*	*P*
**Raw expression values**															
miR-15a-5p	0.88	0.73–1.06	0.19	0.73	0.54–0.98	**0.04**	0.86	0.73–1.00	0.05	1.03	0.80–1.31	0.84	0.14	0.25	0.15
miR-17-3p	0.68	0.54–0.84	**0.0005**	0.92	0.68–1.23	0.57	0.77	0.64–0.91	**0.003**	1.01	0.78–1.31	0.92	0.16	0.11	0.67
miR-193b-5p	0.82	0.70–0.95	**0.01**	0.74	0.54–1.01	0.06	0.82	0.72–0.94	**0.004**	0.92	0.77–1.10	0.34	–c	–	–
miR-204-5p	0.83	0.71–0.96	**0.02**	0.92	0.68–1.23	0.56	0.83	0.71–0.97	**0.02**	0.90	0.71–1.14	0.38	0.86	0.60	0.25
miR-501-5p	0.78	0.63–0.97	**0.03**	0.88	0.63–1.23	0.46	0.81	0.66–0.99	**0.04**	0.96	0.69–1.32	0.78	0.48	0.47	0.84
miR-545-3p	0.87	0.77–1.00	**0.04**	0.87	0.73–1.05	0.15	0.86	0.77–0.96	**0.005**	1.00	0.88–1.14	0.97	0.11	0.17	0.29
miR-592	0.71	0.54–0.93	**0.01**	0.80	0.62–1.01	0.06	0.77	0.67–0.90	**0.0007**	0.81	0.58–1.12	0.21	0.14	0.11	0.82
miR-664-3p	0.87	0.66–1.15	0.32	0.60	0.41–0.87	**0.007**	0.80	0.65–0.99	**0.04**	0.81	0.58–1.13	0.21	0.10	**0.04**	0.31
**Mean-normalized expression values**															
miR-17-3p	0.78	0.63–0.98	**0.03**	1.05	0.83–1.34	0.68	0.89	0.76–1.05	0.16	1.10	0.82–1.48	0.52	0.22	0.09	0.26
miR-155-5p	1.36	1.07–1.71	**0.01**	1.33	1.00–1.77	0.05	1.38	1.15–1.64	**0.0005**	1.05	0.79–1.38	0.74	0.05	0.18	0.18
miR-185-5p	1.45	1.17–1.80	**0.0008**	1.23	0.87–1.74	0.24	1.30	1.10–1.54	**0.003**	0.89	0.67–1.19	0.44	0.06	0.20	0.43
miR-204-5p	0.82	0.68–0.98	**0.03**	1.13	0.87–1.46	0.38	0.90	0.78–1.03	0.13	0.95	0.79–1.13	0.54	0.15	0.07	0.14
miR-449a	1.25	1.03–1.52	**0.02**	0.97	0.73–1.28	0.81	1.15	0.99–1.35	0.07	1.00	0.80–1.26	0.96	0.55	0.89	0.74

a Interaction tests were performed for all patients and for patients with distal (sigmoid and rectum) and proximal (cecum to descending) primary tumor location, separately.

b Hazard ratio is per inter-quartile range expression increase and is adjusted for age, sex, number of metastatic sites, prior adjuvant treatment and primary tumor location.

c miRNA was missing in >10% of samples so interaction was not tested.

Abbreviations: HR, hazard ratio; CI, confidence interval.

Kaplan-Meier curves for TTP according to quartiles of miR-664-3p- and miR-455-5p expression are shown in Figure S2 and S3 in File S1.

### Focused miRNA panel – miRNAs associated with OS

**Figure 1:**
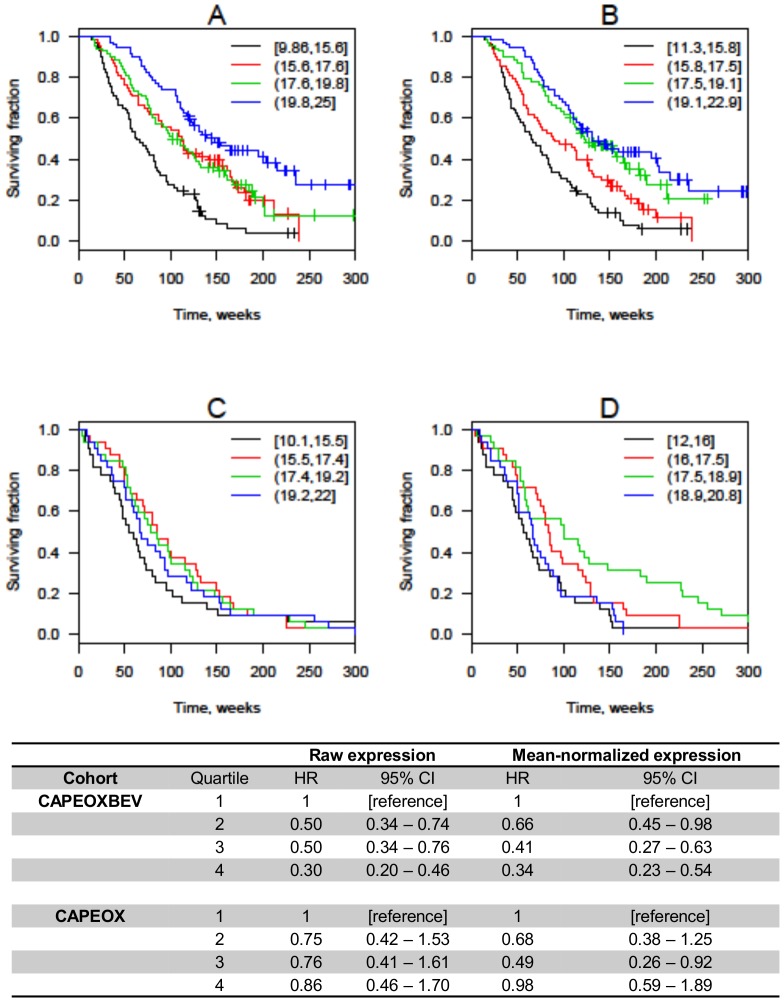
Overall survival according to quartiles of mir-664-3p expression. Kaplan-Meier plots are shown for patients treated with CAPEOXBEV using raw (A) or mean-normalized (B) expression and patients treated with CAPEOX alone using raw (C) and mean-normalized (D) expression. Hazard ratios (HR) are unadjusted and confidence intervals (CI) are calculated using bootstrapping. The expression intervals shown in the upper right-hand corner are 40-C_t_, so higher values correspond to higher expression. Black line = lowest quartile; red line = second quartile; green line = third quartile; blue line = highest quartile.

Twelve miRNAs were significantly associated with OS in one or more of the screening-, validation- and control cohorts (Table 3). Higher miR-664-3p expression was associated with longer OS in the screening and validation cohorts using raw expression: HR 0.64 (CI 0.48–0.86) and 0.60 (CI 0.44–0.82); and normalized expression: HR 0.66 (CI 0.49–0.91) and 0.55 (CI 0.39–0.79). No association between miR-664-3p expression and OS was found in the control cohort. A significant interaction between miR-664-3p expression and bevacizumab effect was observed using both raw- and normalized expression (*P* = 0.02 and *P* = 0.02). Kaplan-Meier plots for OS according to quartiles of miR-664-3p expression are shown in Figure 1.

**Table 3:** Focused miRNA panel – miRNAs significantly associated with overall survival.

	Screening CAPEOXBEV cohort			Validation CAPEOXBEV cohort			Combined CAPEOXBEV cohort			Control CAPEOX cohort			Interaction^a^		
	n = 155			n = 121			n = 276			n = 127			all	distal	proximal
miRNA	HR^b^	95% CI	*P*	HR	95% CI	*P*	HR	95% CI	*P*	HR	95% CI	*P*	*P*	*P*	*P*
**Raw expression values**															
miR-15a-5p	0.80	0.65–0.99	**0.04**	0.87	0.66–1.16	0.34	0.81	0.69–0.97	**0.02**	1.05	0.83–1.34	0.68	0.31	0.41	0.74
miR-196b-5p	0.83	0.69–1.01	0.06	0.66	0.50–0.87	**0.003**	0.77	0.66–0.90	**0.0008**	0.78	0.62–0.98	**0.04**	0.20	0.06	0.23
miR-204-5p	0.76	0.64–0.91	**0.003**	0.88	0.66–1.18	0.38	0.77	0.64–0.93	**0.005**	1.02	0.81–1.28	0.87	0.94	0.80	0.54
miR-338-3p	0.81	0.65–1.00	**0.045**	0.93	0.70–1.24	0.63	0.85	0.72–1.01	0.07	1.01	0.80–1.27	0.92	0.52	0.40	0.68
miR-545-3p	0.85	0.74–0.98	**0.03**	0.95	0.80–1.12	0.53	0.90	0.81–1.01	0.06	1.06	0.95–1.19	0.30	0.39	0.39	0.45
miR-552	0.77	0.61–0.96	**0.02**	0.93	0.68–1.27	0.63	0.77	0.63–0.95	**0.01**	1.02	0.80–1.31	0.85	0.44	0.29	0.76
miR-592	0.69	0.69–0.92	**0.01**	0.76	0.60–0.95	**0.01**	0.73	0.62–0.86	**0.0001**	0.76	0.55–1.05	0.10	0.31	0.33	0.85
miR-664-3p	0.64	0.48–0.86	**0.003**	0.60	0.44–0.82	**0.002**	0.61	0.49–0.75	**<0.0001**	0.85	0.61–1.17	0.31	**0.02**	**0.03**	0.50
**Mean-normalized expression values**															
miR-1	1.01	0.77–1.32	0.96	1.19	0.84–1.69	0.32	1.08	0.87–1.32	0.49	1.36	1.02–1.82	**0.04**	0.98	0.82	0.75
miR-155-5p	1.31	1.03–1.67	**0.03**	1.21	0.91–1.61	0.19	1.23	1.03–1.47	**0.03**	0.96	0.75–1.23	0.76	0.38	0.70	0.30
miR-185-5p	1.49	1.18–1.88	**0.0008**	1.23	0.89–1.72	0.21	1.34	1.12–1.60	**0.001**	0.91	0.70–1.18	0.46	0.09	0.23	0.57
miR-196b-5p	0.89	0.67–1.18	0.43	0.70	0.51–0.96	**0.03**	0.79	0.64–0.97	**0.03**	0.73	0.58–0.93	**0.01**	0.99	0.43	0.09
miR-204-5p	0.75	0.61–0.93	**0.009**	0.95	0.70–1.28	0.71	0.85	0.72–1.01	0.06	1.01	0.85–1.21	0.89	0.43	0.61	0.40
miR-455-5p	1.19	0.95–1.49	0.14	1.27	1.03–1.56	**0.03**	1.24	1.06–1.45	**0.007**	0.99	0.85–1.15	0.91	**0.02**	0.19	0.38
miR-592	0.77	0.59–1.00	0.05	0.76	0.63–0.93	**0.007**	0.77	0.66–0.89	**0.0004**	0.71	0.53–0.96	**0.03**	0.52	0.21	0.35
miR-664-3p	0.66	0.49–0.91	**0.01**	0.55	0.39–0.79	**0.001**	0.60	0.48–0.76	**<0.0001**	0.81	0.59–1.11	0.18	**0.02**	**0.02**	0.45

a Interaction was tested for all patients and for patients with distal (sigmoid to rectum) and proximal (cecum to descending colon) primary tumor location separately.

b Hazard ratio is per inter-quartile range expression increase and is adjusted for age, sex, number of metastatic sites, prior adjuvant treatment and primary tumor location.

Abbreviations: HR, hazard ratio; CI, confidence interval.

**Figure 2:**
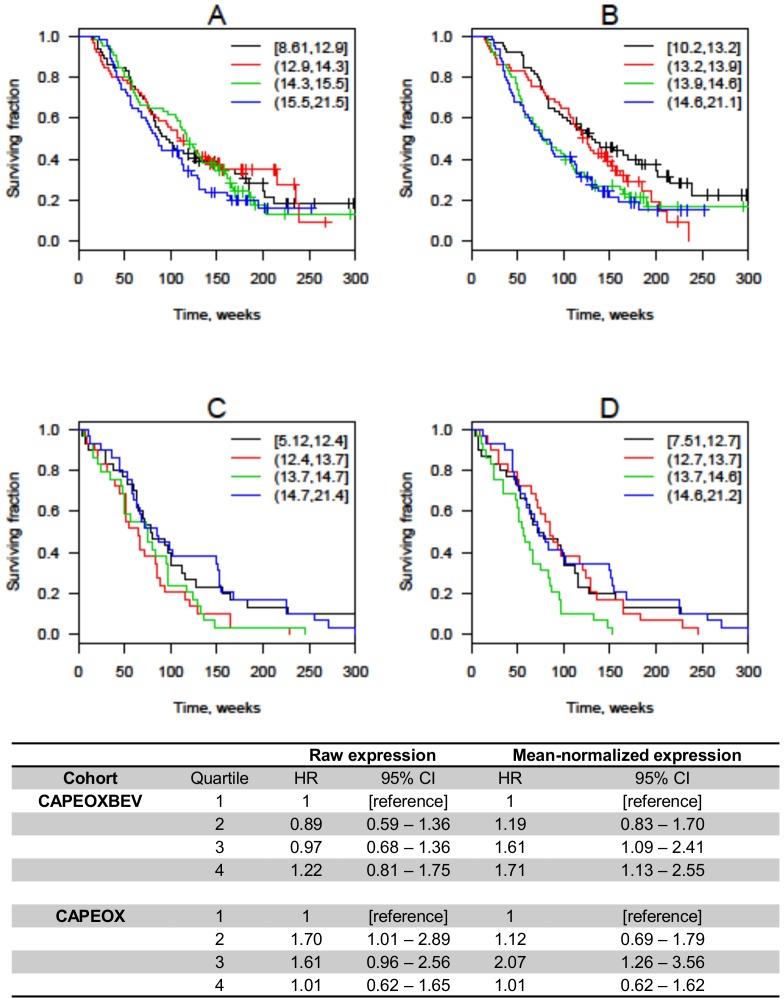
Overall survival according to quartiles of mir-455-5p expression. Kaplan-Meier plots are shown for patients treated with CAPEOXBEV using raw (A) or mean-normalized (B) expression and patients treated with CAPEOX alone using raw (C) and mean-normalized (D) expression. Hazard ratios (HR) are unadjusted and confidence intervals (CI) are calculated using bootstrapping. The expression intervals shown in the upper right-hand corner are 40-C_t_, so higher values correspond to higher expression. Black line = lowest quartile; red line = second quartile; green line = third quartile; blue line = highest quartile.

Higher miR-455-5p expression was associated with shorter OS in the combined bevacizumab-treated cohort when using normalized expression: HR 1.24 (CI 1.06–1.45), but not in the control cohort. There was a significant interaction with bevacizumab effect (*P* = 0.02). Kaplan-Meier plots for OS according to quartiles of miR-455-5p expression are shown in Figure 2.

Higher miR-592 expression was associated with longer OS in the screening- and validation cohorts using raw expression: HR 0.69 (CI 0.69–0.92) and 0.76 (CI 0.60–0.95); and in the validation cohort using normalized expression: HR 0.76 (CI 0.63–0.93), with a similar trend in the screening cohort using normalized expression: HR 0.77 (CI 0.59–1.00). Higher miR-592 expression was also associated with longer OS in the control group when using normalized expression: HR 0.71 (CI 0.53–0.96).

Higher miR-196b-5p expression was associated with longer OS using raw- and normalized expression both in the combined bevacizumab-treated cohort: HR 0.77 (CI 0.66–0.90) and 0.79 (CI 0.64–0.97); and in the control group: HR 0.78 (CI 0.62–0.98) and 0.73 (CI 0.58–0.93).

### Focused miRNA panel – primary tumor location and second line outcome

In analyses stratified for primary tumor location, a significant interaction with bevacizumab effect was seen for miR-664-3p expression in the sigmoid colon and rectum group for both TTP using raw expression and for OS using raw- and normalized expression. Expression levels of all miRNAs according to primary tumor location are shown in Figure S4 in File S1.

In patients continuing bevacizumab in second line, high miR-664-3p expression was associated with longer TTP (HR 0.30, *P* = 0.04) and high miR-455-5p expression was associated with a trend towards shorter TTP (HR 2.72, *P* = 0.09), while no such associations were seen in patients that did not continue bevacizumab (Figure S5 in File S1).

### MiRNA *in situ* hybridization

**Figure 3:**
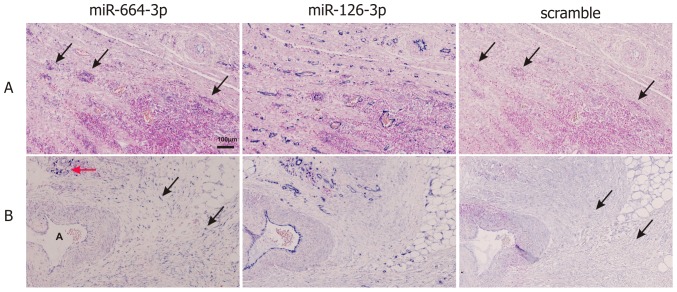
MiR-664-3p *in situ* hybridization (ISH). Panels A and B show examples of miR-664-3p ISH in infiltrating lymphocytes (A) and fibroblasts (B). Consecutive sections were stained with LNA probes against miR-664-3p, miR-126-3p and a scramble sequence. MiR-664-3p ISH signal is seen in infiltrating lymphocytes (A, arrows; B, red arrow) and in fibroblasts (B, black arrows), whereas no ISH signal is obtained with scramble probe. A strong ISH signal is seen in endothelial cells with the positive control probe miR-126-3p. The “A” in panel B indicates an artery.

An intense miR-664-3p ISH signal, primarily with a cytoplasmic localization, was seen in subpopulations of tumor-infiltrating lymphocytes, fibroblasts, and endothelial cells located at the invasive border (Figure 3). A weak miR-664-3p ISH signal was seen in tumor epithelial cells, but a similar staining was observed with the scramble probe, suggesting an unspecific binding of the probe to these cells. MiR-455-5p ISH signal was found in some lymphocyte-like stromal cells in half of the samples, while tumor epithelial cells were negative (Figure S6 in File S1). No ISH signal was obtained with the probes against miR-185, miR-449a or miR-592. Positive control probes for miR-21-5p and miR-126-3p showed moderate to intense staining in fibroblasts and endothelial cells, respectively, in all cases.

### Target prediction

Table S2 in File S1 shows the 20 highest ranked predicted gene targets for miR-196b-5p, miR-455-5p, miR-592, and miR-664-3p, and references published articles regarding the function and expression level of these miRNAs in cancer.

## Discussion

This is the first comprehensive study of miRNAs as predictive biomarkers for bevacizumab effectiveness in CRC. Of the 22 miRNAs selected from the screening study, miR-664-3p and miR-455-5p showed the greatest potential as predictive biomarkers for bevacizumab effectiveness.

The association between miR-664-3p and OS differed significantly between patients treated with bevacizumab and patients treated with chemotherapy alone: Increasing miR-664-3p expression in primary CRC tissue was associated with longer OS in both cohorts of patients treated with bevacizumab combined with CAPEOX but not in the cohort treated with chemotherapy alone. Increasing miR-664-3p expression was also associated with longer TTP in patients treated with bevacizumab, but the interaction test was only significant in the subgroup of patients with sigmoid colon- and rectal primary tumors. We previously hypothesized that this subgroup of patients could be more likely to derive benefit from treatment with bevacizumab than patients with more proximal primary tumors [20]. MiR-664-3p expression was also higher in these patients than in patients with more proximal primary tumors (Figure S4 in File S1). In the small cohort of patients with available second line outcome data, high miR-664-3p expression was also associated with a longer TTP only in patients continuing bevacizumab, supporting a connection between miR-664-3p expression and bevacizumab effectiveness.

We observed high expression of miR-664-3p in stromal cells, including endothelial cells, which is in accordance with a role for miR-664-3p in angiogenesis. Very little data have been published about this miRNA (Table S2 in File S1). Interestingly, among the top predicted targets of miR-664-3p are neuroligin 1 (NLGN1), MDGA2, and gephyrin, which are all involved in the same synaptogenic process in the nervous system [30], [31]. Recently, neuroligin and its binding partner neurexin have been shown to be widely expressed in the vascular system and involved in angiogenesis [30]. Overexpression of neuroligin 1 in endothelial cells grown in a tumorigenic environment increased angiogenesis, and knockdown of neurexin reduced fibroblast growth factor 2-induced angiogenesis [32]. In a zebra fish embryo model of angiogenesis, inhibition of VEGF-A or neuroligin caused similar magnitudes of vascular defects, but inhibition of both resulted in a more than additive anti-angiogenic effect [33]. Hypothetically, the impact of miR-664-3p expression on outcome may thus be explained by its downregulation of the neuroligin system and the resulting synergy with VEGF-A inhibition by bevacizumab.

Increasing miR-455-5p expression was associated with shorter OS in the combined bevacizumab treated cohort while no such association was observed in the cohort treated with chemotherapy alone. We identified high expression of this miRNA in lymphocyte-like cells located in the stroma around the cancer cells. MiR-455-5p has been reported to be dysregulated in cancer; however, no validated targets have been identified (Table S2 in File S1).

Increasing expression of both miR-196b-5p and miR-592 was associated with longer OS in all three cohorts, with similar effect estimates. Both these miRNAs have been shown to be downregulated in CRCs with deficient mismatch repair (dMMR) [34]. Higher miR-592 expression has been shown to be associated with improved OS in patients receiving salvage anti-EGFR treatment and higher miR-196b-5p expression has been linked to response to neo-adjuvant 5-FU and radiotherapy in patients with locally advanced rectal cancer [35], [36]. MiR-592 expression has been reported to be higher in left-sided compared to right-sided CRCs [37], which we also found in our study. We could not stain our tissue sections for miR-592, but expression of both miR-592 and miR-196b-5p has previously been shown to be 2.5–3.7 fold higher in CRC epithelium than in CRC stroma [38]. The function of miR-592 has not been described. MiR-196b-5p is dysregulated in many malignancies, has been related to cancer prognosis, and targets c-myc, ERG, MEIS1, FAS, ABL1, BCL-2 and several HOX genes (Table S2 in File S1).

There are important limitations to consider regarding our results. We studied retrospectively identified cohorts from different time periods, which increases the risk of bias, since differences other than the treatments used could exist between the cohorts. We used mean expression for normalization, but since the miRNAs used for calculating the mean were related to outcome, this is sub-optimal. Even though the association with outcome for some of the miRNAs was identified in two or three independent cohorts, the predictive effect related to bevacizumab remains un-validated. We did not correct for multiple testing, but miR-664-3p would still be significantly associated with OS in the validation cohort, even after correcting for the 22 miRNAs tested. Also, effect estimates were similar in the cohorts, indicating a non-random association. Among the strengths of our study are the large sample size, the initial comprehensive screening study, the use of three independent cohorts, and randomization of purification- and miRNA expression analysis order.

In conclusion, this is the first study to examine the potential for miRNA expression in primary tumors to predict benefit of bevacizumab in patients with mCRC. We have identified miR-664-3p and miR-455-5p as possible predictive biomarkers for bevacizumab. MiR-592 and miR-196b-5p were predictive of outcome both with and without bevacizumab and these could be prognostic biomarkers or biomarkers related to chemotherapy effectiveness. These findings need validation in independent cohorts – preferably from randomized trials and using stable miRNA normalizers –before they can be implemented in clinical decision making. Elucidation of the cellular origins and biological functions of these miRNAs is warranted.

## Supporting Information

File S1 This file contains supplementary methods, supplementary Table S1 and S2, and supplementary Figure S1–S6.
(PDF)
