# Supplementary material for: Tissue MicroRNAs as Predictors of Outcome in Patients with Metastatic Colorectal Cancer Treated with First Line Capecitabine and Oxaliplatin with or without Bevacizumab
Source: PLoS One. 2014 Oct 15;9(10):e109430. doi: 10.1371/journal.pone.0109430 (PMC4198118; doi:10.1371/journal.pone.0109430)
Supplement: File S1 — This file contains supplementary methods, supplementary Table S1 and S2, and supplementary Figure S1–S6. (PDF) [file pone.0109430.s001.pdf]

Supplementary methods

Patients, data extraction, and end points

Inclusion criteria were biopsy-confirmed adenocarcinoma of the colon or rectum and first line palliative treatment with CAPEOXBEV or CAPEOX. Exclusion criteria were other coexisting malignancy or endocrine histology. Patients in the CAPEOX cohort were also not allowed to have been treated with BEV in later lines. The ten departments of oncology from which we included patients encompassed all of the departments in the country which identified CAPEOXBEV as one of their preferred first line treatments for patients with mCRC. The randomized study from which we partly included our control cohort was a study of chronomodulated versus standard-dosing of CAPEOX, i.e. all patients received CAPEOX in the same dose [1]. Treatment was given as infusional oxaliplatin 130 mg/m^2 on day 1, capecitabine 2000 mg/m^2 daily days 1–14, +/- infusional bevacizumab 7.5 mg/kg on day 1, repeated every 3 weeks.

Data were extracted from individual patient records and electronic databases at each hospital using a standardized case report form. Pathology data and survival status were extracted from national databases using the unique Civil Registration System number assigned to every Danish citizen.

Disease evaluation was done every three to four cycles using computed tomography (CT) scans and the Response Evaluation Criteria In Solid Tumors (RECIST) 1.0 criteria [2]. Date of disease progression was defined as the date of an evaluation CT scan showing progression according to the RECIST 1.0 criteria or, in a few cases, by other clinical signs of progression, if a diagnosis of clinical progression was stated unequivocally in the patient record. The end point time to disease progression (TTP) was measured from initiation of treatment to disease progression. If patients died without evidence of disease progression they were censored at the last known date of non-

progression. If patients died without having any disease evaluations they were excluded from the TTP analyses.

Primary tumor location was registered as 1: cecum and ascending colon, 2: right flexure and transverse colon, 3: left flexure and descending colon, 4: sigmoid- and rectosigmoid colon, or 5: rectum.

More than 98% of patients were White.

MiRNA expression analysis

Briefly, the procedure utilized for the miRNeasy FFPE kit RNA purification was as follows. Samples were deparaffinized and then lysed with proteinase K digestion followed by heat treatment. After centrifugation, the supernatant was recovered and treated with DNase. After mixing with buffer and ethanol, part of the mixture was transferred to an RNeasy MinElute spin column where total RNA was bound. After washing, the RNA was eluted and normalized to 70 ng/µl either manually (screening cohort) or using an automated procedure (validation and control cohorts).

The purity and concentration of RNA was assessed by absorbance spectrometry on a NanoDrop 8000 (Thermo Fisher Scientific, Waltham, Massachusetts, USA). Samples with a 260nm/280nm-ratio below 1.8 were discarded and new sections from the corresponding tissue block were cut and purified.

Samples were stored at -80 °C until they were used for miRNA expression analysis.

The procedure utilized for the Applied Biosystems Human LDA Card Set array analyses was as follows. RNA was transcribed into cDNA in 2 multiplex reactions each containing 3 µl of the small

RNA preparation, corresponding to 200 ng total RNA, and either Megaplex RT Primer Pool A or Pool B, and using the TaqMan® MicroRNA Reverse Transcription Kit in a total reaction volume of 16 µl. Prior to loading the arrays, a 12-cycle pre-amplification reaction was performed using 2.5 µl cDNA in a 25-µl reaction and using Megaplex PreAmp Primers Pool A or B. Each of the arrays was loaded with 1/50 of the preamplification reaction which was mixed with TaqMan Gene Expression Master Mix in a total reaction volume of 800 µl and run on the 7900HT Fast Real-Time PCR System.

The same procedures were executed for the custom LDA card as for the large array, except for 8 different samples being loaded onto each card instead of one.

MiRNA in situ hybridization

Sequence and melting temperature for the targeted probes were as follows.

miR-185-5p (TCAGGAACTGCCTTTCTCTCCA, RNA T_m 85°C),

miR-449a (ACCAGCTAACAATACACTGCCA, RNA T_m 82°C),

miR-455-5p (CGATGTAGTCCAAAGGCACATA, RNA T_m 83°C),

miR-592 (ACATCATCGCATATTGACACAA, RNA T_m 84°C),

miR-664-3p (TGTAGGCTGGGGATAAATGAATA, RNA T_m 81°C),

miR-21-5p (TCAACATCAGTCTGATAAGCTA, RNA T_m 83°C), and

miR-126-3p (CATTATTACTCACGGTACGA, RNA T_m 84°C).

A 22-mer scrambled LNA probe with a random sequence (TGTAACACGTCTATACGCCCA, RNA T_m 87°C) was included as a negative control. All LNA oligos were 5, 6-carboxyfluorescein

(FAM)-labeled at both the 5'- and 3'-ends. Briefly, 6 µm thick tissue sections were mounted on glass slides and deparaffinized and then placed in a Tecan Freedom Evo automated hybridization instrument *(*Tecan, Männedorf, Switzerland*)* in which the following steps were performed: proteinase-K treatment (25 µg/ml at 37°C for 8 min.), pre-hybridization (at 57°C for 15 min.), hybridization with FAM-labeled LNA probes (40 nM/ml probe at 57°C for 60 min.), stringent washes with SSC buffers (at 57°C for at least 33 min.), blocking reagent (PBS containing 2% Sheep serum, 1% BSA and 0.05% Tween-20), alkaline phosphatase-conjugated anti-FAM, enzymatic development that precipitates the blue NBT-BCIP substrate, and nuclear fast red counterstain. The sections were finally dehydrated and mounted. All LNA probes were HPLC purified.

In total, twenty patient samples were studied using ISH.

Statistical analysis

Differences in characteristics between cohorts were tested using Wilcoxon Mann-Whitney test for continuous variables, Fisher's exact test for categorical variables, and log rank test for time to event variables.

<u>Screening study</u>

The C_ts (before subtracting from 40) were adjusted according to the spike-in miRNA value (ath-miR-159a). That is, for each C_t-value in a given subject we subtract 0.28 × (spike-in - 22) from the C_t-value, since the spike-in is supposed to be approximately 22 (data not shown).

The calculations relating miRNA expression to outcome were done for both raw-, quantile-normalized [3] and mean-normalized data sets. The number of missing values within each miRNA was counted before the normalization. If certain miRNAs had more than 45% missing values they were removed in the quantile-normalized data set, because of the normalization function's

requirements. Mean-normalization was performed by subtracting the mean of the 120 miRNAs with the lowest mean C_t from the C_t of all miRNAs. This is done sample-wise.

Both $P < 0.001$ and a threshold found by cross validation [4] were used for selecting which miRNAs to include in the multivariate analyses.

The criteria used for selecting miRNAs from the screening study for further investigation were:

1. Retained in multivariate models after backwards selection using at least 2 types of expression data (raw, quantile-normalized, mean-normalized) for TTP or OS
2. OR retained in a multivariate model after backwards selection using 1 type of expression data for TTP or OS and within the top 35 most significantly correlated to TTP or OS using both raw- and quantile-normalized expression data
3. OR within the top 5 most significantly correlated to TTP or OS univariately using both raw- and quantile-normalized expression data

The confidence intervals in the unadjusted analyses that are shown in Figure 1 and 2 and in Figure S2 and S3 were computed using BCa bootstrapping [5] with R=999 bootstrap replications.

Table S1. MiRNAs retained in one or more multivariate models after backwards selection or top-5 most significant univariately

		Multivariate model in which a given miRNA was retained – by end point and data type									
		TTP					OS				
**MiRNA name**[a]	**Mature miRNA sequence**[a]	**Raw**	**Raw CV**	**Quan**	**Quan CV**	**Mean**	**Raw**	**Raw CV**	**Quan**	**Quan CV**	**Mean**
hsa-miR-1	UGGAAUGUAAAGAAGUAUGUAU					x					
hsa-miR-15a-5p	UAGCAGCACAUAAUGGUUUGUG								x	x	
hsa-miR-17-3p	ACUGCAGUGAAGGCACUUGUAG				x	x					
hsa-miR-22-3p	AAGCUGCCAGUUGAAGAACUGU	x	x			x				x	
hsa-miR-29a-5p[b]	ACUGAUUUCUUUUGGUGUUCAG					x					
hsa-miR-29b-3p	UAGCACCAUUUGAAAUCAGUGUU	x	x			x		x			x
hsa-miR-145-3p	GGAUUCCUGGAAAUACUGUUCU	x	x	x	x						
hsa-miR-148a-3p[b]	UCAGUGCACUACAGAACUUUGU								x	x	
hsa-miR-155-5p	UUAAUGCUAAUCGUGAUAGGGGU								x		
hsa-miR-181a-5p[b]	AACAUUCAACGCUGUCGGUGAGU							x			
hsa-miR-185-5p	UGGAGAGAAAGGCAGUUCCUGA			x							
hsa-miR-193b-5p	CGGGGUUUUGAGGGCGAGAUGA	x	x	x	x	x					
hsa-miR-196b-5p	UAGGUAGUUUCCUGUUGUUGGG						x	x		x	x
hsa-miR-204-5p	UUCCCUUUGUCAUCCUAUGCCU				x	x			x	x	x
hsa-miR-214-5p	UGCCUGUCUACACUUGCUGUGC					x					x
hsa-miR-338-3p	UCCAGCAUCAGUGAUUUUGUUG								x		
hsa-miR-382-5p	GAAGUUGUUCGUGGUGGAUUCG		x				x	x	x	x	
hsa-miR-449a	UGGCAGUGUAUUGUUAGCUGGU									x	x
hsa-miR-449b-5p[b]	AGGCAGUGUAUUGUUAGCUGGC								x		
hsa-miR-455-5p	UAUGUGCCUUUGGACUACAUCG							x			
hsa-miR-497-5p	CAGCAGCACACUGUGGUUUGU					x					
hsa-miR-501-5p	AAUCCUUUGUCCCUGGGUGAGA					x					
hsa-miR-545-3p	UCAGCAAACAUUUAUUGUGUGC								x	x	x
hsa-miR-552-3p	AACAGGUGACUGGUUAGACAA								x		x
hsa-miR-592[c]	UUGUGUCAAUAUGCGAUGAUGU										
hsa-miR-664-3p	UAUUCAUUUAUCCCCAGCCUACA				x	x					

[a] www.mirbase.org, accessed October 22 2013

[b] These miRNAs were not selected for further study

[c] In univariate analyses, miR-592 was significantly correlated to OS with the second lowest p-value both using raw- and quantile-normalized expression

Abbreviations: TTP, time to disease progression; OS, overall survival; Raw, non-normalized raw values; HR, hazard ratio per inter-quartile range increase; Quan, quantile-normalized values; CV, threshold for inclusion determined by cross-validation (ref); mean, mean-normalized values

Table S2. Predicted targets and publications regarding identified miRNAs

miR-664-3p

Top 20 predicted target genes

ZNF423, CTD-2162K18.4, ***NLGN1,*** *C18orf34, PRTG, SLC10A2, C6orf106,* ***MDGA2,*** *EBF2, PLP2, IRF2, RSBN1, MYOG,* ***GPHN,*** *AHCTF1, KLF12, CT47A4, ZMYND11, CT47A8, CT47A10*

Publications

Cell/tissue type	Function/expression level	Author	Year
Hepatocellular carcinoma	Upregulated; targets MAT1A; increases tumor growth, invasion, and metastasis	Yang [6]	2013
**Myocardial endothelial cells**	**Upregulated in endothelial cells from diabetic mice with impaired angiogenesis**	**Wang [7]**	**2009**

miR-455-5p

Top 20 predicted target genes

GDAP2, EHD4, MYLIP, BRD1, KDSR, LYPD3, MOB4, SESN3, TMEM30A, ZNF385A, GABRB2, KCNJ2, NDUFAF4, SOCS3, MIPOL1, RPS6KB1, TAOK1, LUC7L3, ZFYVE26, SUZ12

Publications

Cell/tissue type	Function/expression level	Author	Year
Basal cell carcinoma	Upregulated	Sand [8]	2012
Endometrial cancer	Downregulated	Hiroki [9]	2010
Laryngeal cancer	Upregulated	Saito [10]	2013
Medullary thyroid carcinoma	Downregulated	Hudson [11]	2013
Murine cardiac myocytes	Downregulated by ATF6, which is activated by ischemic stress	Belmont [12]	2012
**Murine macrophages**	**Upregulated in response to *Candida albicans***	**Monk [13]**	**2010**
**Skeletal muscle cells**	**Upregulated by proinflammatory TWEAK cytokine**	**Panguluri [14]**	**2010**

miR-592

Top 20 predicted target genes

PAFAH1B1, AKAP6, ROCK1, LRRC4C, ATP5A1, C14orf102, CHGB, STX16, CAND1, PTPRD, ZSWIM6, NFIA, SCGB2A2, RP11-67H2.1, TTC29, KLHL29, EMR2, USP9X, CEACAM3, PRUNE2

Publications

Cell/tissue type	Function/expression level	Author	Year
**Colorectal cancer**	**Downregulated in dMMR and upregulated in pMMR (versus normal epithelium)**	**Sarver [15]**	**2009**
**Colorectal cancer**	**2.5-fold higher in cancer epithelium than cancer stroma**	**Nishida [16]**	**2012**
**Colorectal cancer**	**3.96-fold higher in left colon and rectum versus right colon**	**Schee [17]**	**2013**
**Colorectal cancer**	**Higher in patients with disease control versus progression as best response on treatment with anti-EGFR**	**Mosakhani [18]**	**2012**
Colorectal cancer	Upregulated in doxorubicin-resistant cell line	Qu [19]	2013
Hepatocellular carcinoma	Downregulated in HBV-associated HCC	Wang [20]	2012

miR-196b-5p

Top 20 predicted target genes

HOXA7, HOXC8, SLC9A6, WIPF2, HAND1, HOXA9, PSMD11, CASK, GATA6, HOXA5, CCDC47, BCAT1, LRP1B, CCNJ, NR6A1, VSNL1, ZBTB26, HOXB7, COL1A2, GDF3

Publications

Cell/tissue type	Function/expression level	Author	Year
**Colorectal cancer**	**Downregulated in dMMR and unchanged in pMMR (versus normal epithelium)**	**Sarver [15]**	**2009**
**Colorectal cancer**	**3.7-fold higher in cancer epithelium than cancer stroma**	**Nishida [16]**	**2012**
**Rectal cancer**	**Upregulated in cancers that responded to neo-adjuvant 5-fluorouracil and radiation**	**Svoboda [21]**	**2012**
Breast cancer	Inhibited HOXC8 and inhibited migration and metastases	Li [22]	2010
Cervical cancer	Downregulated; high expression associated with improved disease-free survival; targets *HOXB7*	How [23]	2013
Endometriotic cyst stromal cells	Overexpression lowered c-myc and Bcl-2 mRNA expression	Abe [24]	2013
Endothelial cells	Upregulated in tumor-associated Ecs; upregulated by VEGF treatment; suppression led to increased HOXD10; Suppression decreased EC profileration and migration	Plummer [25]	2013
Esophageal cancer	Upregulated in tumor from patient with good prognosis	Zhao [26]	2013
Esophageal cancer	Upregulated	Liu [27]	2013
Gastric cancer	Upregulated	Tsai [28]	2010
Gastric cancer	Downregulated by ETS2 and induces epithelial-mesenchymal transition	Liao [29]	2012
Gastric cancer	Upregulated	An [30]	2013
Gastric cancer	Upregulated; high expression was associated with poor survival; expression correlated positively with HOXA10 expression	Lim [31]	2013
Glioblastoma	Upregulated; high miR-196b-5p was associated with shorter survival	Guan [32]	2010
Glioblastoma	Upregulated; high miR-196b-5p was associated with longer survival	Lakomy [33]	2011
Glioblastoma	Overexpression increases proliferation; High miR-196b-5p was associated with shorter survival	Ma [34]	2012
Hepatocellular carcinoma	Upregulated	Shen [35]	2013
Leukemia	Upregulated; increases proliferation and decreases differentiation; associated with HOX-gene activation	Popovic [36]	2009
Leukemia	Downregulated; inhibited c-myc	Bhatia [37]	2010
Leukemia	Overexpression inhibited ERG transcription factor	Coskun [38]	2011
Leukemia	Targets both oncogenes HOXA9/MEIS1 and tumor suppressor gene FAS	Li [39]	2012
Leukemia	Downregulated	Bhatia [40]	2011
Leukemia	Downregulated; targets *HOXA9* and *ABL1*	Liu [41]	2013
Leukemia	High expression was associated with shorter overall survival	Diaz-Beyá [42]	2013
Osteosarcoma	Upregulated	Namløs [43]	2012
Pancreatic cancer	Upregulated	Yu [44]	2012
Pancreatic cancer	Upregulated	Schultz [45]	2012
Peripheral nerve s heath tumors	Upregulated in malignant versus benign lesions	Masliah-Planchon [46]	2013
Prostate cancer	Epigenetically downregulated	Hulf [47]	2011

Genes and publications in bold are discussed in the manuscript.

[a] Genes are ranked according to the most likely targets identified using the DIANA-microT-CDS (v5.0) algorithm (reference in manuscript)

[c] Three additional HOX genes are predicted as targets among the top 100 target (HOXB1, HOXB6, and HOXB7)

Abbreviations: MREs, miRNA recognition elements

Figure S1. CONSORT diagram

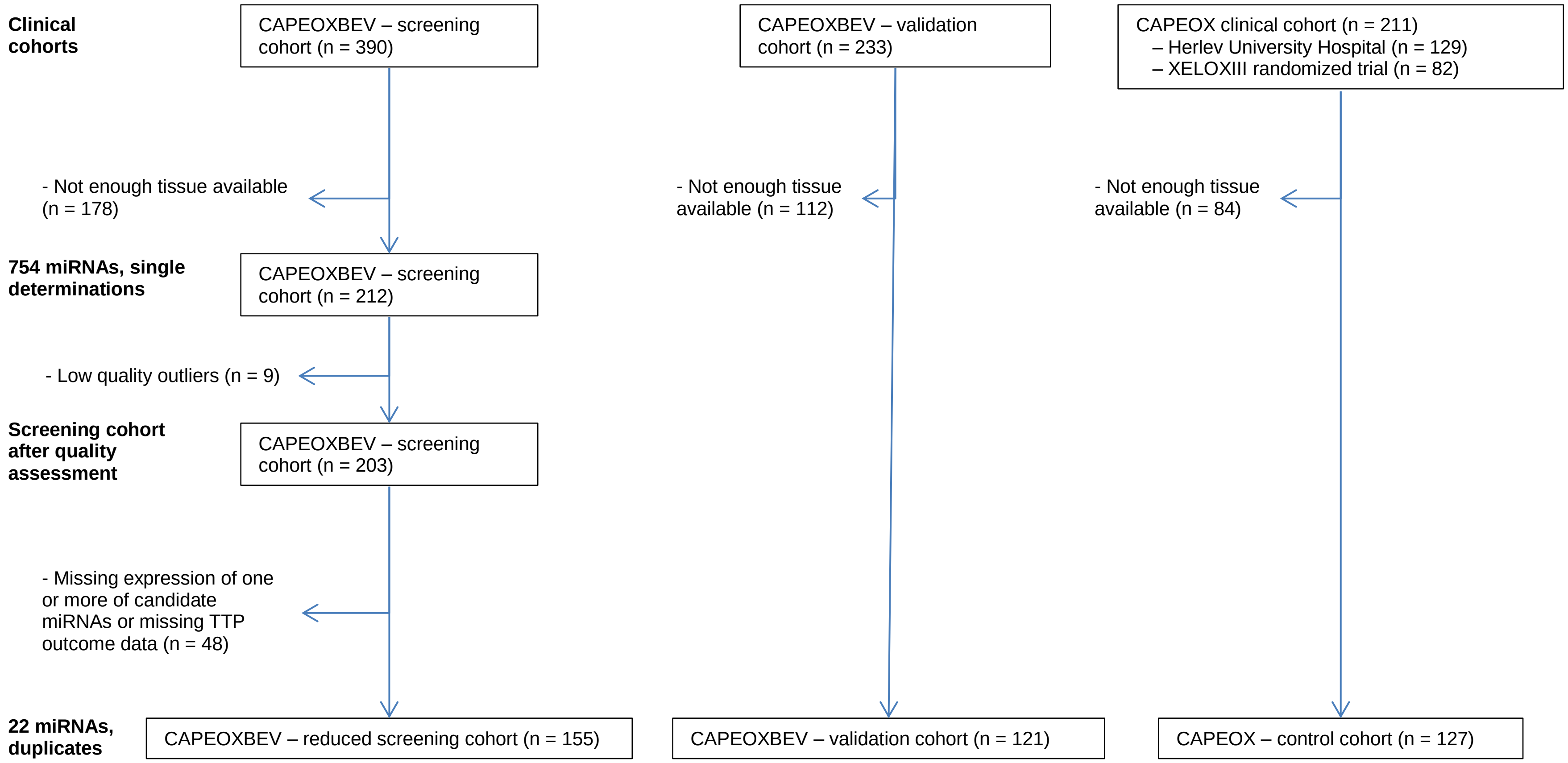

Figure S2. Time to disease progression according to quartiles of mir-664-3p expression

Kaplan Meier plots are shown for patients treated with CAPEOXBEV using raw (A) or mean-normalized (B) expression and patients treated with CAPEOX alone using raw (C) and mean-normalized (D) expression. Hazard ratios (HR) are unadjusted and confidence intervals (CI) are calculated using bootstrapping. The expression intervals shown in the upper right-hand corner are 40-C_t, so higher values correspond to higher expression. Black line = lowest quartile; red line = second quartile; green line = third quartile; blue line = highest quartile.

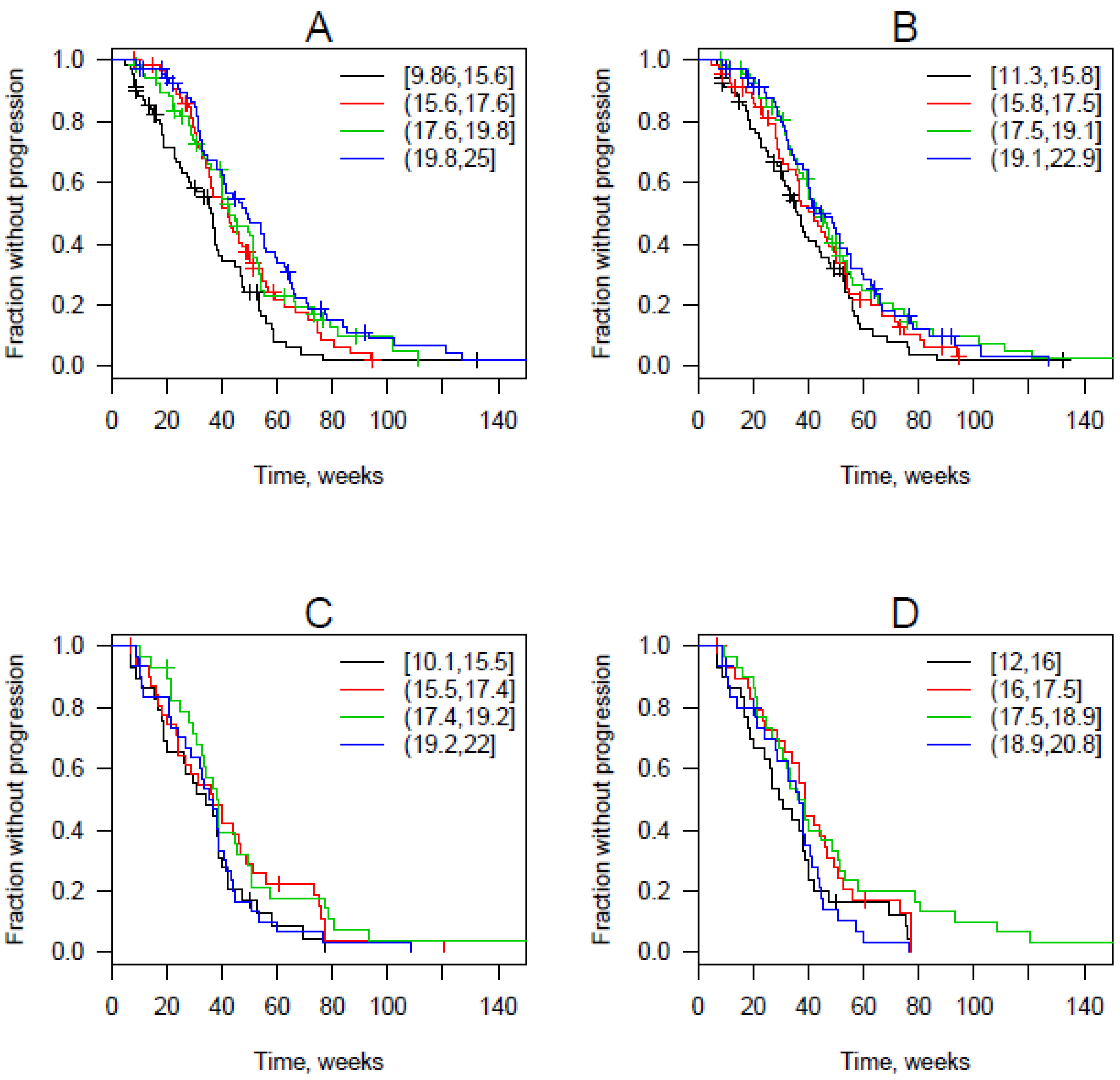

		Raw expression		Mean-normalized expression	
**Cohort**	**Quartile**	**HR**	**95% CI**	**HR**	**95% CI**
**CAPEOXBEV**	1	1	[reference]	1	[reference]
	2	0.67	0.46 – 1.01	0.81	0.57 – 1.30
	3	0.61	0.42 – 0.98	0.68	0.46 – 1.02
	4	0.50	0.34 – 0.78	0.67	0.47 – 1.00
**CAPEOX**	1	1	[reference]	1	[reference]
	2	0.69	0.39 – 1.19	0.71	0.42 – 1.19
	3	0.61	0.33 – 1.04	0.52	0.30 – 1.05
	4	0.91	0.49 – 1.52	0.99	0.57 – 1.57

Figure S3. Time to disease progression according to quartiles of mir-455-5p expression

Kaplan Meier plots are shown for patients treated with CAPEOXBEV using raw (A) or mean-normalized (B) expression and patients treated with CAPEOX alone using raw (C) and mean-normalized (D) expression. Hazard ratios (HR) are unadjusted and confidence intervals (CI) are calculated using bootstrapping. The expression intervals shown in the upper right-hand corner are 40-C_t, so higher values correspond to higher expression. Black line = lowest quartile; red line = second quartile; green line = third quartile; blue line = highest quartile.

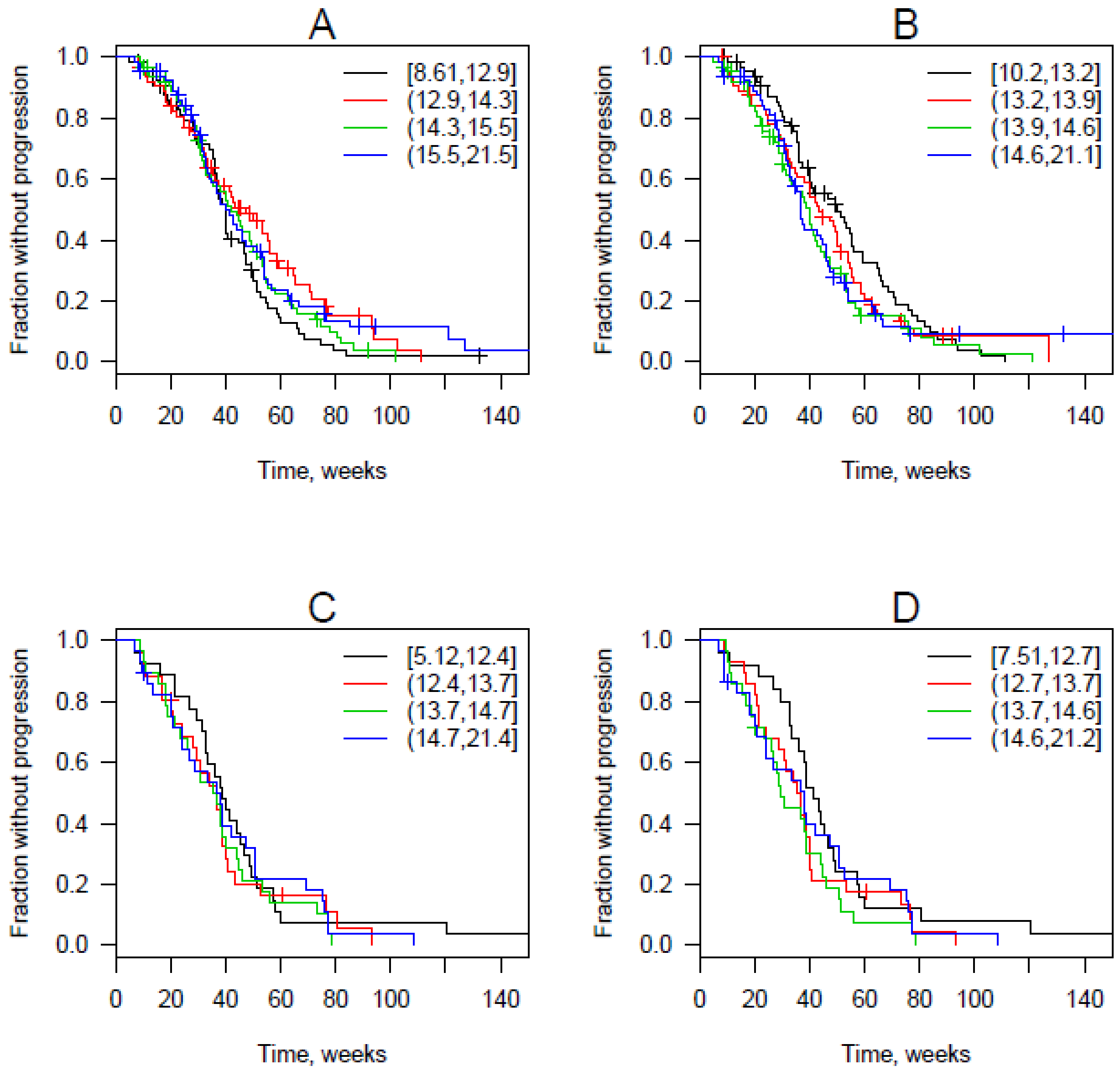

		Raw expression		Mean-normalized expression	
**Cohort**	**Quartile**	**HR**	**95% CI**	**HR**	**95% CI**
**CAPEOXBEV**	1	1	[reference]	1	[reference]
	2	0.72	0.50 – 1.03	1.13	0.80 – 1.58
	3	0.88	0.64 – 1.22	1.38	0.99 – 1.91
	4	0.76	0.51 – 1.12	1.25	0.81 – 1.83
**CAPEOX**	1	1	[reference]	1	[reference]
	2	1.11	0.62 – 2.03	1.27	0.74 – 2.21
	3	1.19	0.74 – 1.98	1.48	0.88 – 2.63
	4	1.03	0.66 – 1.87	1.17	0.67 – 2.02

Figure S4. MiRNA expression level in cancer, inflammatory bowel disease and in normal bowel

Group	Number of samples
1 = cecum and ascending colon	87
2 = right flexure and transverse colon	26
3 = left flexure and descending colon	22
4 = sigmoid- and rectosigmoid colon	131
5 = rectum	137
Colitis ulcerosa	10
Crohn’s disease	10
Normal colon	10
Normal rectum	10

miR-1

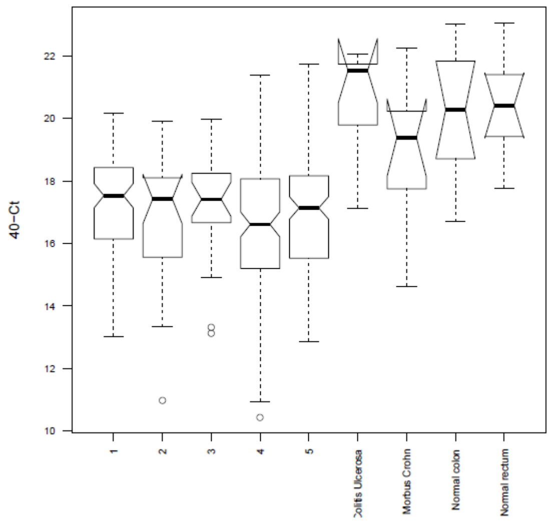

miR-15a-5p

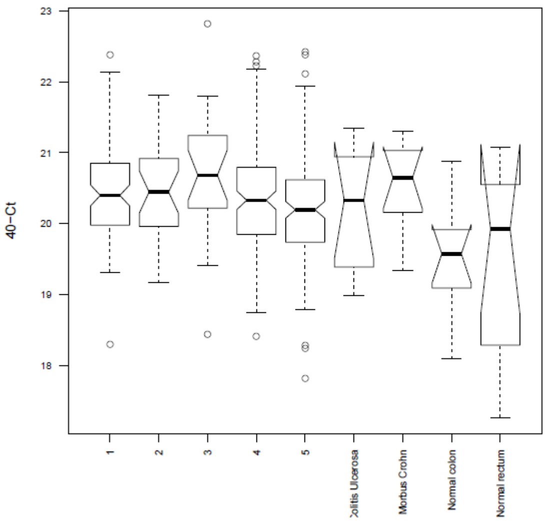

miR-17-3p

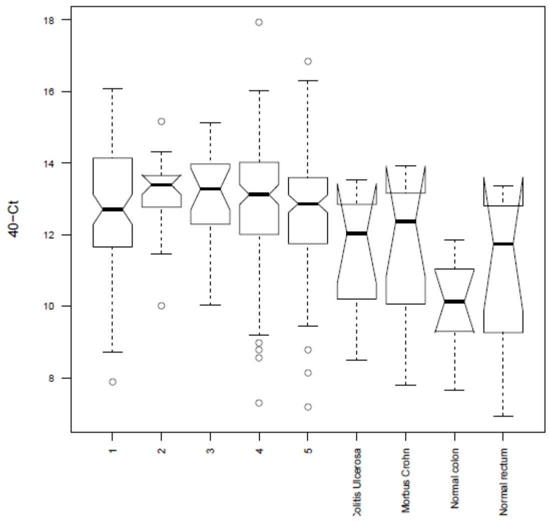

miR-22-3p

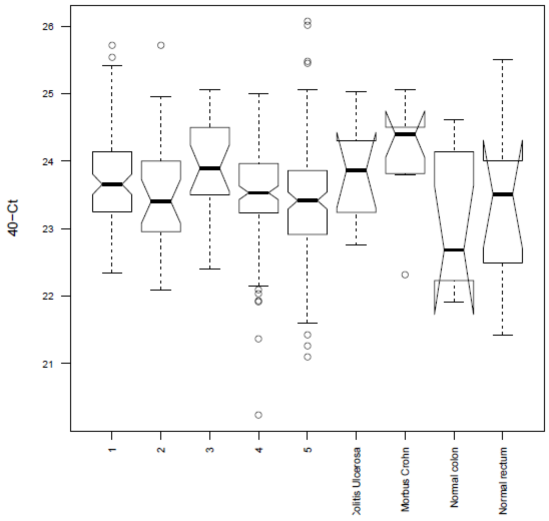

miR-29b-3p

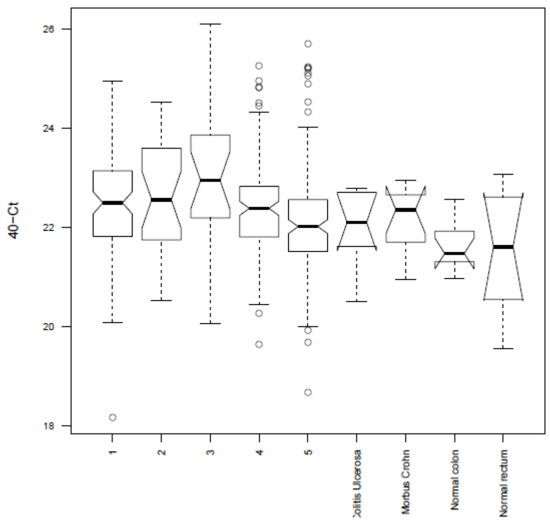

miR-145-3p

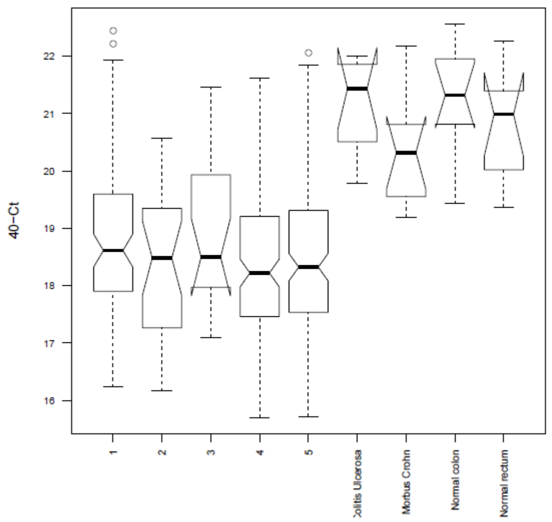

miR-155-5p

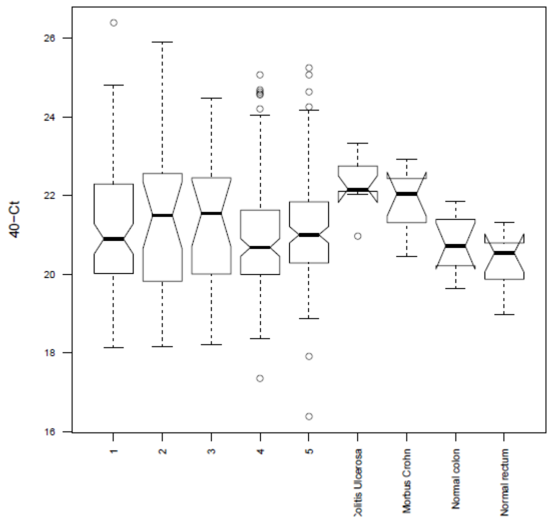

miR-185-5p

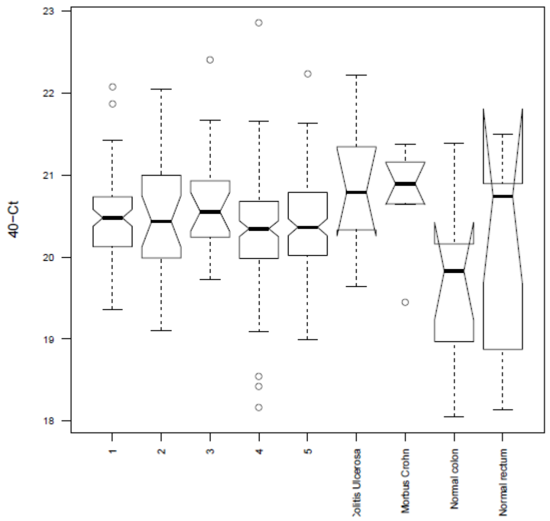

miR-193b-5p

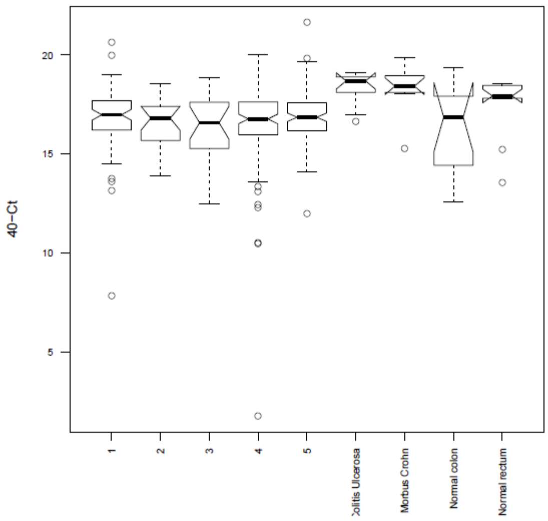

miR-196-5p

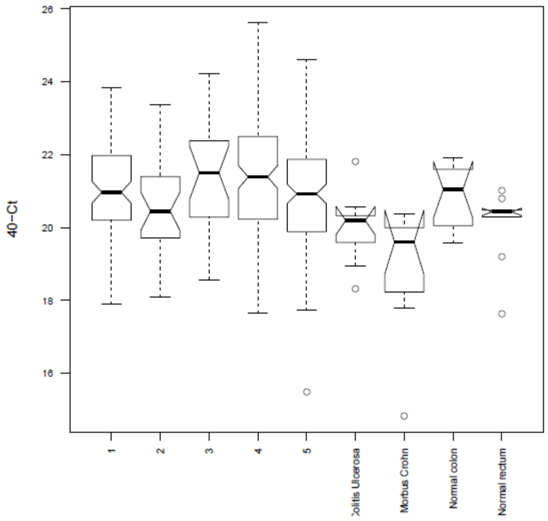

miR-204-5p

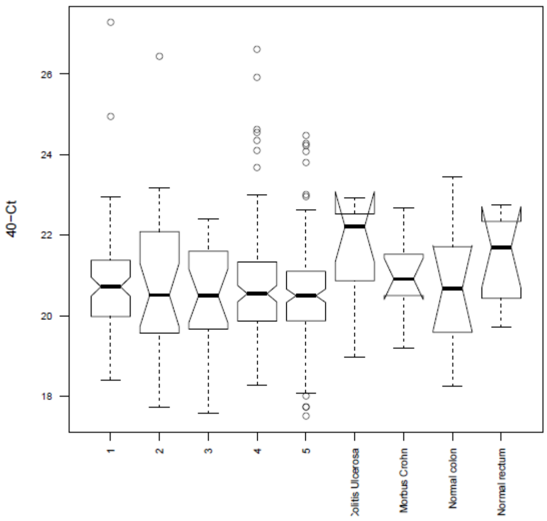

miR-214-5p

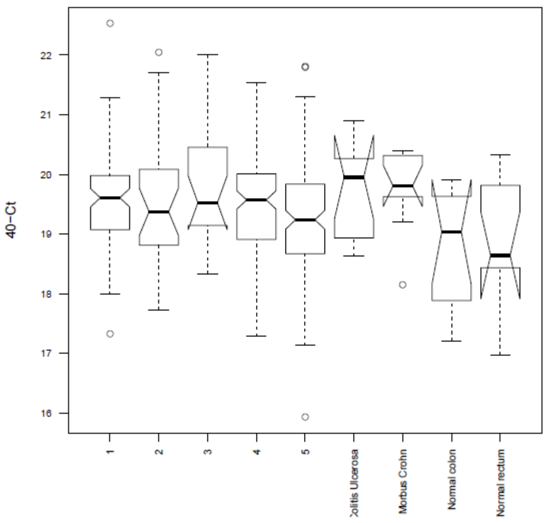

miR-338-3p

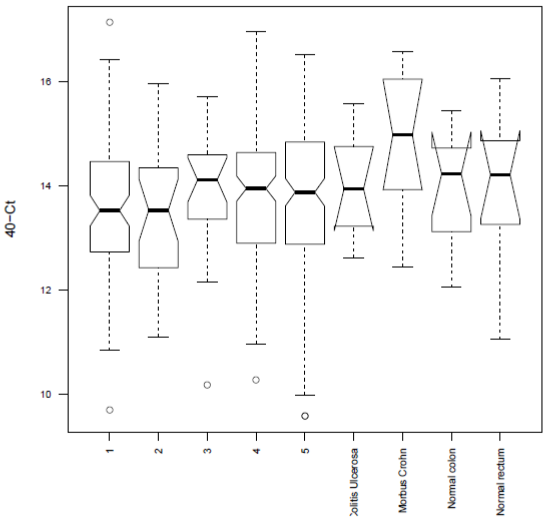

miR-382-5p

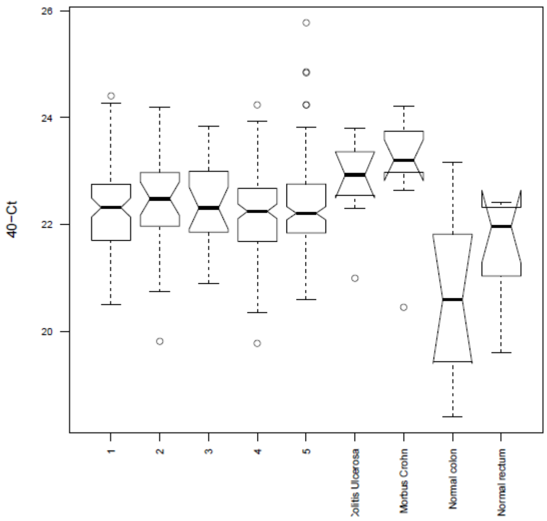

miR-449a

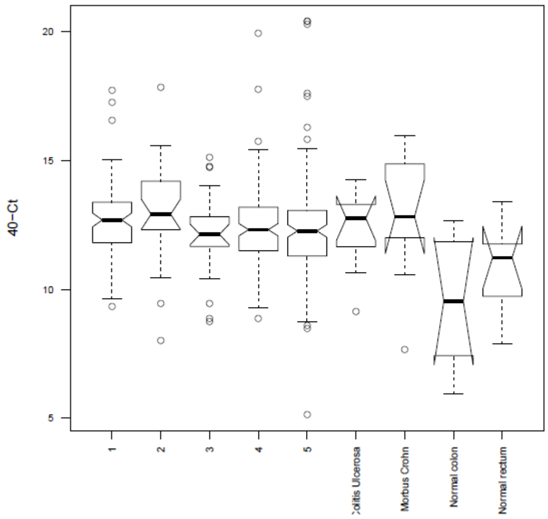

miR-455-5p

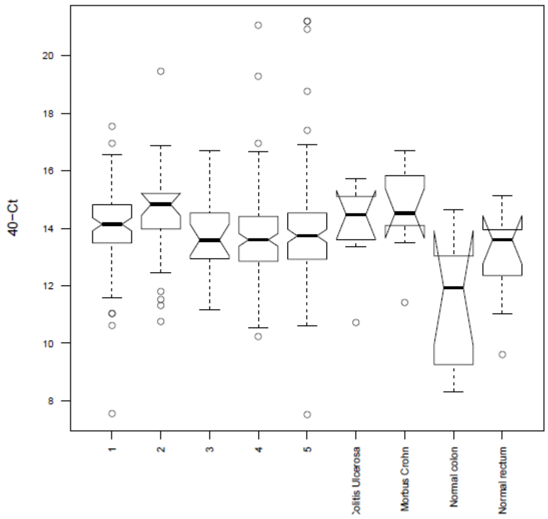

miR-497-5p

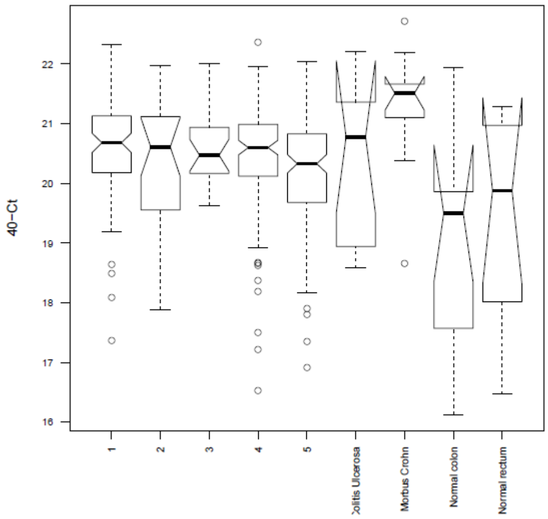

miR-501-5p

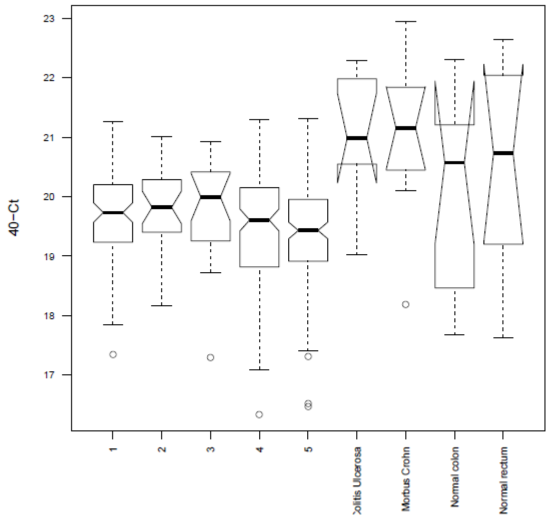

miR-545-3p

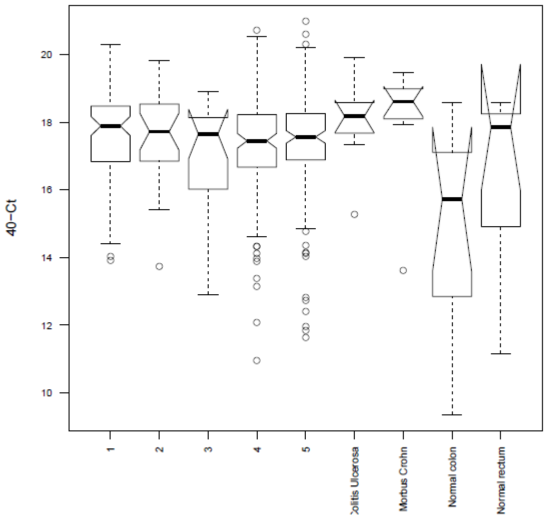

miR-552-3p

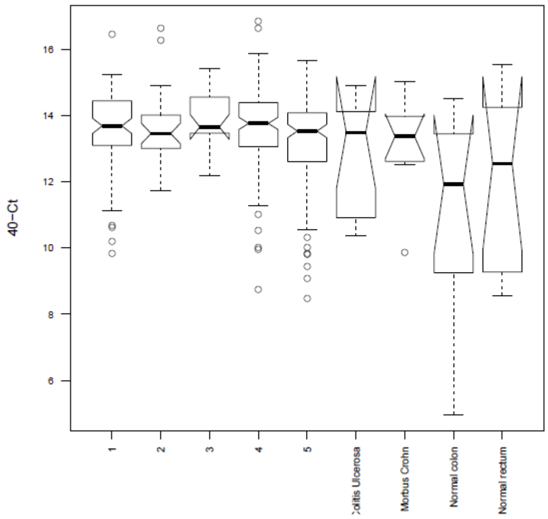

miR-592

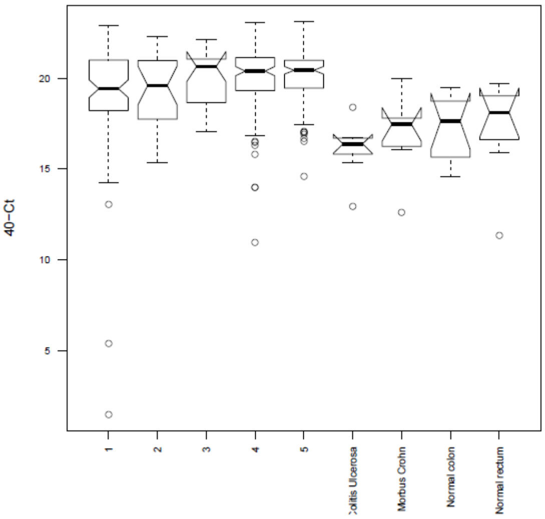

miR-664-3p

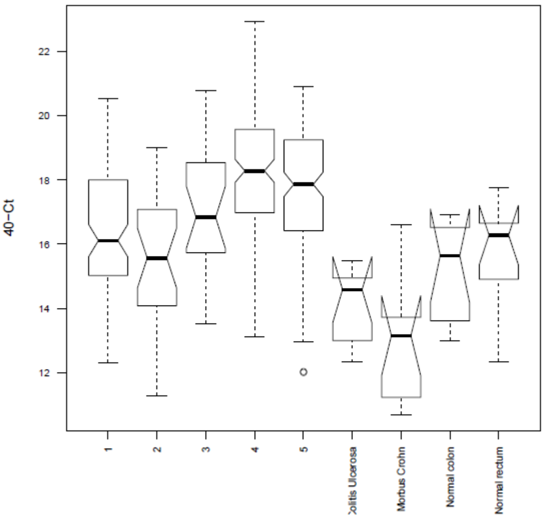

Figure S5. Second line irinotecan-containing regimen time to disease progression (TTP): association with miRNA expression

<u>Kaplan-Meier survival plots</u>

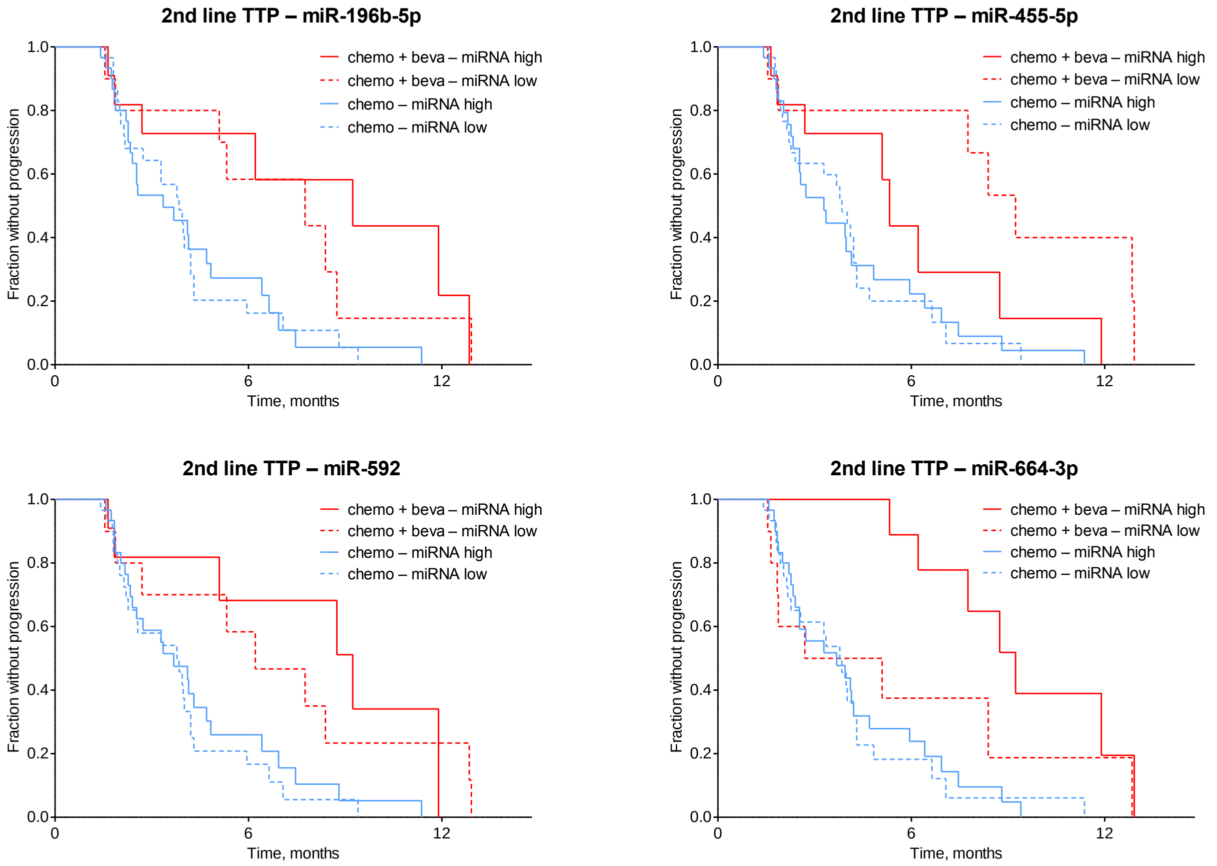

miRNA	Chemotherapy alone (n=60)<br>Median TTP (months)<br>miRNA high (n=30)	miRNA low (n=30)	HR	P^a	Chemotherapy + bevacizumab (n=21)<br>Median TTP (months)<br>miRNA high (n=11)	miRNA low (n=10)	HR	P^a
miR-196b-5p	3.4	3.8	0.95	0.85	9.2	7.8	0.82	0.71
miR-455-5p	3.3	3.8	0.99	0.98	5.3	9.2	2.72	0.09
miR-592	3.7	3.8	0.80	0.45	9.2	6.2	0.80	0.69
miR-664-3p	3.7	3.8	0.97	0.90	9.2	3.9	0.30	**0.04**

Abbreviations: HR, hazard ratio
[a] log rank test

Figure S6. MiR-455-5p *in situ* hybridization (ISH)

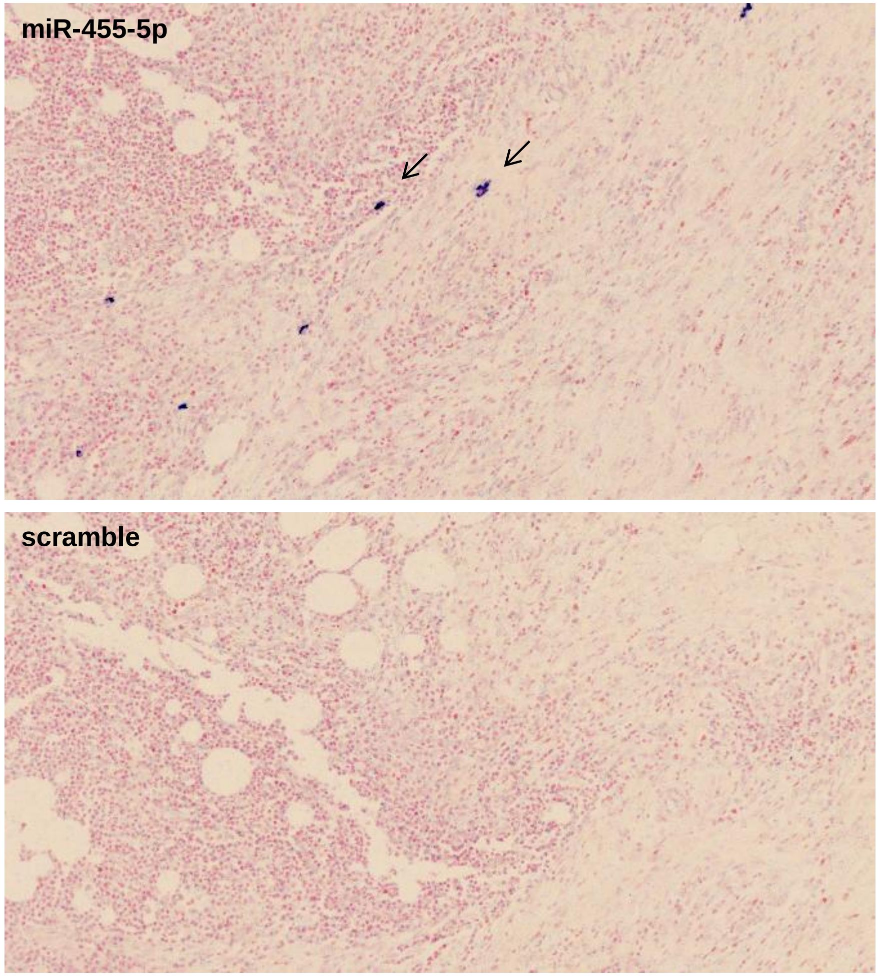

Example of miR-455-5p ISH in a colon cancer specimen. Consecutive sections were stained with LNA probes against miR-455-5p and a scramble sequence. miR-455-5p ISH signal is restricted to a few lymphocyte-like cells (examples indicated by arrows), whereas no ISH signal is obtained with the scramble probe.

References

1. Qvortrup C, Jensen BV, Fokstuen T, Nielsen SE, Keldsen N, et al. (2010) A randomized study comparing short-time infusion of oxaliplatin in combination with capecitabine XELOX(30) and chronomodulated XELOX(30) as first-line therapy in patients with advanced colorectal cancer. Ann Oncol 21: 87-91.
2. Therasse P, Arbuck SG, Eisenhauer EA, Wanders J, Kaplan RS, et al. (2000) New guidelines to evaluate the response to treatment in solid tumors. European Organization for Research and Treatment of Cancer, National Cancer Institute of the United States, National Cancer Institute of Canada. J Natl Cancer Inst 92: 205-216.
3. Bolstad BM, Irizarry RA, Astrand M, Speed TP (2003) A comparison of normalization methods for high density oligonucleotide array data based on variance and bias. Bioinformatics 19: 185-193.
4. Bovelstad HM, Nygard S, Storvold HL, Aldrin M, Borgan O, et al. (2007) Predicting survival from microarray data--a comparative study. Bioinformatics 23: 2080-2087.
5. Efron B (1987) Better Bootstrap Confidence Intervals. Journal of the American Statistical Association 82: 171-185.
6. Yang H, Cho ME, Li TW, Peng H, Ko KS, et al. (2013) MicroRNAs regulate methionine adenosyltransferase 1A expression in hepatocellular carcinoma. J Clin Invest 123: 285-298.
7. Wang XH, Qian RZ, Zhang W, Chen SF, Jin HM, et al. (2009) MicroRNA-320 expression in myocardial microvascular endothelial cells and its relationship with insulin-like growth factor-1 in type 2 diabetic rats. Clin Exp Pharmacol Physiol 36: 181-188.
8. Sand M, Skrygan M, Sand D, Georgas D, Hahn SA, et al. (2012) Expression of microRNAs in basal cell carcinoma. Br J Dermatol 167: 847-855.

9. Hiroki E, Akahira J, Suzuki F, Nagase S, Ito K, et al. (2010) Changes in microRNA expression levels correlate with clinicopathological features and prognoses in endometrial serous adenocarcinomas. Cancer Sci 101: 241-249.

10. Saito K, Inagaki K, Kamimoto T, Ito Y, Sugita T, et al. (2013) MicroRNA-196a is a putative diagnostic biomarker and therapeutic target for laryngeal cancer. PLoS One 8: e71480.

11. Hudson J, Duncavage E, Tamburrino A, Salerno P, Xi L, et al. (2013) Overexpression of miR-10a and miR-375 and downregulation of YAP1 in medullary thyroid carcinoma. Exp Mol Pathol 95: 62-67.

12. Belmont PJ, Chen WJ, Thuerauf DJ, Glembotski CC (2012) Regulation of microRNA expression in the heart by the ATF6 branch of the ER stress response. J Mol Cell Cardiol 52: 1176-1182.

13. Monk CE, Hutvagner G, Arthur JS (2010) Regulation of miRNA transcription in macrophages in response to Candida albicans. PLoS One 5: e13669.

14. Panguluri SK, Bhatnagar S, Kumar A, McCarthy JJ, Srivastava AK, et al. (2010) Genomic profiling of messenger RNAs and microRNAs reveals potential mechanisms of TWEAK-induced skeletal muscle wasting in mice. PLoS One 5: e8760.

15. Sarver AL, French AJ, Borralho PM, Thayanithy V, Oberg AL, et al. (2009) Human colon cancer profiles show differential microRNA expression depending on mismatch repair status and are characteristic of undifferentiated proliferative states. BMC Cancer 9: 401.

16. Nishida N, Nagahara M, Sato T, Mimori K, Sudo T, et al. (2012) Microarray analysis of colorectal cancer stromal tissue reveals upregulation of two oncogenic miRNA clusters. Clin Cancer Res 18: 3054-3070.

17. Schee K, Lorenz S, Worren MM, Gunther CC, Holden M, et al. (2013) Deep Sequencing the MicroRNA Transcriptome in Colorectal Cancer. PLoS One 8: e66165.

18. Mosakhani N, Lahti L, Borze I, Karjalainen-Lindsberg ML, Sundstrom J, et al. (2012) MicroRNA profiling predicts survival in anti-EGFR treated chemorefractory metastatic colorectal cancer patients with wild-type KRAS and BRAF. Cancer Genet 205: 545-551.

19. Qu J, Zhao L, Zhang P, Wang J, Xu N, et al. (2013) MicroRNA-195 chemosensitizes colon cancer cells to the chemotherapeutic drug doxorubicin by targeting the first binding site of BCL2L2 mRNA. J Cell Physiol.

20. Wang W, Zhao LJ, Tan YX, Ren H, Qi ZT (2012) Identification of deregulated miRNAs and their targets in hepatitis B virus-associated hepatocellular carcinoma. World J Gastroenterol 18: 5442-5453.

21. Svoboda M, Sana J, Fabian P, Kocakova I, Gombosova J, et al. (2012) MicroRNA expression profile associated with response to neoadjuvant chemoradiotherapy in locally advanced rectal cancer patients. Radiat Oncol 7: 195.

22. Li Y, Zhang M, Chen H, Dong Z, Ganapathy V, et al. (2010) Ratio of miR-196s to HOXC8 messenger RNA correlates with breast cancer cell migration and metastasis. Cancer Res 70: 7894-7904.

23. How C, Hui AB, Alajez NM, Shi W, Boutros PC, et al. (2013) MicroRNA-196b regulates the homeobox B7-vascular endothelial growth factor axis in cervical cancer. PLoS One 8: e67846.

24. Abe W, Nasu K, Nakada C, Kawano Y, Moriyama M, et al. (2013) miR-196b targets c-myc and Bcl-2 expression, inhibits proliferation and induces apoptosis in endometriotic stromal cells. Hum Reprod 28: 750-761.

25. Plummer PN, Freeman R, Taft RJ, Vider J, Sax M, et al. (2013) MicroRNAs regulate tumor angiogenesis modulated by endothelial progenitor cells. Cancer Res 73: 341-352.

26. Zhao BS, Liu SG, Wang TY, Ji YH, Qi B, et al. (2013) Screening of microRNA in patients with esophageal cancer at same tumor node metastasis stage with different prognoses. Asian Pac J Cancer Prev 14: 139-143.

27. Liu SG, Qin XG, Zhao BS, Qi B, Yao WJ, et al. (2013) Differential expression of miRNAs in esophageal cancer tissue. Oncol Lett 5: 1639-1642.

28. Tsai KW, Hu LY, Wu CW, Li SC, Lai CH, et al. (2010) Epigenetic regulation of miR-196b expression in gastric cancer. Genes Chromosomes Cancer 49: 969-980.

29. Liao YL, Hu LY, Tsai KW, Wu CW, Chan WC, et al. (2012) Transcriptional regulation of miR-196b by ETS2 in gastric cancer cells. Carcinogenesis 33: 760-769.

30. An J, Pan Y, Yan Z, Li W, Cui J, et al. (2013) MiR-23a in amplified 19p13.13 loci targets metallothionein 2A and promotes growth in gastric cancer cells. J Cell Biochem 114: 2160-2169.

31. Lim JY, Yoon SO, Seol SY, Hong SW, Kim JW, et al. (2013) Overexpression of miR-196b and HOXA10 characterize a poor-prognosis gastric cancer subtype. World J Gastroenterol 19: 7078-7088.

32. Guan Y, Mizoguchi M, Yoshimoto K, Hata N, Shono T, et al. (2010) MiRNA-196 is upregulated in glioblastoma but not in anaplastic astrocytoma and has prognostic significance. Clin Cancer Res 16: 4289-4297.

33. Lakomy R, Sana J, Hankeova S, Fadrus P, Kren L, et al. (2011) MiR-195, miR-196b, miR-181c, miR-21 expression levels and MGMT methylation status are associated with clinical outcome in glioblastoma patients. Cancer Sci.

34. Ma R, Yan W, Zhang G, Lv H, Liu Z, et al. (2012) Upregulation of miR-196b confers a poor prognosis in glioblastoma patients via inducing a proliferative phenotype. PLoS One 7: e38096.

35. Shen J, Wang S, Zhang YJ, Kappil MA, Chen Wu H, et al. (2012) Genome-wide aberrant DNA methylation of microRNA host genes in hepatocellular carcinoma. Epigenetics 7: 1230-1237.

36. Popovic R, Riesbeck LE, Velu CS, Chaubey A, Zhang J, et al. (2009) Regulation of mir-196b by MLL and its overexpression by MLL fusions contributes to immortalization. Blood 113: 3314-3322.

37. Bhatia S, Kaul D, Varma N (2010) Potential tumor suppressive function of miR-196b in B-cell lineage acute lymphoblastic leukemia. Mol Cell Biochem 340: 97-106.

38. Coskun E, von der Heide EK, Schlee C, Kuhnl A, Gokbuget N, et al. (2011) The role of microRNA-196a and microRNA-196b as ERG regulators in acute myeloid leukemia and acute T-lymphoblastic leukemia. Leuk Res 35: 208-213.

39. Li Z, Huang H, Chen P, He M, Li Y, et al. (2012) miR-196b directly targets both HOXA9/MEIS1 oncogenes and FAS tumour suppressor in MLL-rearranged leukaemia. Nat Commun 3: 688.

40. Bhatia S, Kaul D, Varma N (2011) Functional genomics of tumor suppressor miR-196b in T-cell acute lymphoblastic leukemia. Mol Cell Biochem 346: 103-116.

41. Liu Y, Zheng W, Song Y, Ma W, Yin H (2013) Low expression of miR-196b enhances the expression of BCR-ABL1 and HOXA9 oncogenes in chronic myeloid leukemogenesis. PLoS One 8: e68442.

42. Diaz-Beya M, Brunet S, Nomdedeu J, Tejero R, Diaz T, et al. (2013) MicroRNA expression at diagnosis adds relevant prognostic information to molecular categorization in patients with intermediate-risk cytogenetic acute myeloid leukemia. Leukemia.

43. Namlos HM, Meza-Zepeda LA, Baroy T, Ostensen IH, Kresse SH, et al. (2012) Modulation of the osteosarcoma expression phenotype by microRNAs. PLoS One 7: e48086.

44. Yu J, Li A, Hong SM, Hruban RH, Goggins M (2012) MicroRNA alterations of pancreatic intraepithelial neoplasias. Clin Cancer Res 18: 981-992.

45. Schultz NA, Werner J, Willenbrock H, Roslind A, Giese N, et al. (2012) MicroRNA expression profiles associated with pancreatic adenocarcinoma and ampullary adenocarcinoma. Mod Pathol 25: 1609-1622.

46. Masliah-Planchon J, Pasmant E, Luscan A, Laurendeau I, Ortonne N, et al. (2013) MicroRNAome profiling in benign and malignant neurofibromatosis type 1-associated nerve sheath tumors: evidences of PTEN pathway alterations in early NF1 tumorigenesis. BMC Genomics 14: 473.

47. Hulf T, Sibbritt T, Wiklund ED, Bert S, Strbenac D, et al. (2011) Discovery pipeline for epigenetically deregulated miRNAs in cancer: integration of primary miRNA transcription. BMC Genomics 12: 54.
